# M. tuberculosis Ser/Thr Protein Kinase D Phosphorylates an Anti-Anti–Sigma Factor Homolog

**DOI:** 10.1371/journal.ppat.0030049

**Published:** 2007-04-06

**Authors:** Andrew E Greenstein, Jason A MacGurn, Christina E Baer, Arnold M Falick, Jeffery S Cox, Tom Alber

**Affiliations:** 1 Department of Molecular and Cell Biology, University of California, Berkeley, California, United States of America; 2 Department of Microbiology and Immunology, University of California San Francisco, San Francisco, California, United States of America; 3 Biophysics Graduate Group, University of California, Berkeley, California, United States of America; 4 Howard Hughes Medical Institute Mass Spectrometry Laboratory, University of California, Berkeley, California, United States of America; University of Washington, United States of America

## Abstract

Receptor Ser/Thr protein kinases are candidates for sensors that govern developmental changes and disease processes of *Mycobacterium tuberculosis (Mtb),* but the functions of these kinases are not established. Here, we show that *Mtb* protein kinase (Pkn) D overexpression alters transcription of numerous bacterial genes, including Rv0516c, a putative anti-anti–sigma factor, and genes regulated by sigma factor F. The PknD kinase domain directly phosphorylated Rv0516c, but no other sigma factor regulator, in vitro. In contrast, the purified PknB and PknE kinase domains phosphorylated distinct sigma regulators*.* Rather than modifying a consensus site, PknD phosphorylated Rv0516c in vitro and in vivo on Thr2 in a unique N-terminal extension. This phosphorylation inhibited Rv0516c binding in vitro to a homologous anti-anti–sigma factor, Rv2638. These results support a model in which signals transmitted through PknD alter the transcriptional program of *Mtb* by stimulating phosphorylation of a sigma factor regulator at an unprecedented control site.

## Author Summary

Many bacteria, including *Mycobacterium tuberculosis (Mtb),* sense the environment using a family of signaling proteins called Ser/Thr protein kinases (STPKs), but the functions of these sensors are not well understood. This study shows that the *Mtb* protein kinase (Pkn) D STPK attaches a phosphate group to one and only one member of a family of regulators of “alternative” sigma factors, which activate sets of genes in numerous bacteria. Phosphorylation of the regulator at an unprecedented position abolished binding in vitro to a putative partner. Remarkably, increasing PknD activity in *Mtb* not only strongly activated the gene encoding the specific regulatory protein phosphorylated by PknD, but also altered the expression of genes controlled by an alternative sigma factor. By providing evidence for a mechanistic link between PknD and gene regulation, this work supports a new model in which STPKs in numerous microorganisms transduce environmental signals by controlling expression of specific groups of genes. Thus, certain bacterial STPKs may orchestrate aspects of the coordinate control of gene expression essential for adaptation in the environment and in host infections.

## Introduction

*Mycobacterium tuberculosis (Mtb)* is among the world's most harmful pathogens, causing approximately two million deaths annually [1]. In addition to the emergence of multi-drug–resistant strains, *Mtb* evades current therapeutics by shifting from active infection to a persistent, metabolically dormant state [2]. This transition exemplifies the distinctive *Mtb* life cycle, which encompasses unique developmental adaptations to distinct environments [3]. Little is known about the signaling mechanisms that mediate the biochemical changes that initiate and maintain the stages of *Mtb* development.

Candidate regulators of *Mtb* development include receptor Ser/Thr protein kinases (STPKs) that modulate intracellular events in response to external stimuli. In eukaryotes, homologous STPKs sense environmental cues and transduce signals that regulate virtually all aspects of cell physiology. The *Mtb* genome encodes 11 such Hanks-type (also called “eukaryotic-like”) STPKs, including nine putative transmembrane receptor kinases [4]. Although the activating stimuli for these kinases have not been identified, the extracellular C-terminal sensor domains include a β-propeller interaction motif, a PASTA repeat thought to bind cell wall structures, and a redox-sensitive DsbG homolog [5–8]. The intracellular, N-terminal kinase domains structurally resemble eukaryotic homologs, and similar receptor STPKs are widely distributed in bacterial genera. The first reported bacterial STPK substrates include pThr-binding forkhead-associated (FHA) domains [9], metabolic enzymes [10], and apparent regulators of cell division [11,12], but the mechanisms of signaling in vivo are not established. Genetic studies suggest that two of the 11 *Mtb* STPKs are essential for growth [13] and that the STPKs regulate characteristics such as cell shape [11], virulence [14], and nitrogen balance [15]. Identifying the intracellular targets of *Mtb* STPKs is essential to understanding their mechanistic roles in *Mtb* biology.

A second class of bacterial Ser/Thr kinases, the anti–sigma factors, regulates gene expression by controlling alternative sigma factors [16]. Alternative sigma factors, such as sigma B (SigB) and sigma F (SigF) in *Bacillus subtilis,* mediate transcriptional responses to environmental cues by binding RNA polymerase and mediating promoter recognition. Work on B. subtilis has established the paradigm by which complex regulatory cascades influence alternative sigma factor activity (reviewed by Hughes and Mathee [16]). Anti–sigma factor proteins (e.g., RsbW) directly sequester cognate alternative sigma factors and prevent RNA polymerase binding. Anti-anti–sigma factors (e.g., RsbV) relieve this transcriptional repression by binding the anti–sigma factor. The anti–sigma factors phosphorylate anti-anti–sigma factors on a conserved Ser or Thr, and this modification promotes dissociation of the complex.

This basic regulatory organization is recapitulated for multiple layers in which paralogs of anti–sigma factors and anti-anti–sigma factors switch partners and ultimately determine the concentration of the active sigma factor [17]. In this “partner switching” mechanism, anti–sigma factor paralogs play two distinct roles. Some anti–sigma factors (e.g., RsbW) antagonize transcription by directly sequestering alternative sigma factors. In contrast, other anti–sigma factors (e.g., RsbT) act upstream to stimulate transcription by binding and activating the master “environmental sensing” phosphatase (RsbU in B. subtilis [18]). This phosphatase reactivates anti-anti–sigma factors, which bind the cognate anti–sigma factor, thus increasing the concentration of free sigma factor. Environmental cues affect the phosphorylation state of upstream anti-anti–sigma factor paralogs (such as RsbS and the RsbRA-D proteins), and these proteins form a complex (termed the “stressosome”) that binds the positive regulator of the phosphatase [19]. The central role of Ser/Thr phosphorylation in anti-anti–sigma factor regulation and the established role of eukaryotic kinases in gene regulation led us to test the hypothesis that the eukaryotic-like STPKs may impinge on transcription regulated by alternative sigma factors.

Here, we demonstrate that increasing the activity of the PknD STPK in *Mtb* resulted in specific phosphorylation of a single anti-anti–sigma factor homolog, Rv0516c. Simultaneously, the *Rv0516c* gene was activated and transcription of genes regulated by the SigF alternative sigma factor was coordinately altered. PknD phosphorylated Rv0516c at a novel site, Thr2, distinct from conserved Ser/Thr phosphorylation sites in the anti-anti–sigma factor family. Thr2 phosphorylation abolished binding to another anti-anti–sigma factor. These results demonstrate that PknD phosphorylates a putative sigma factor regulator in *Mtb,* alters binding of a cognate regulator, and, by a mechanism that has not been determined, changes the expression of SigF-dependent genes.

## Results

**Figure 1:**
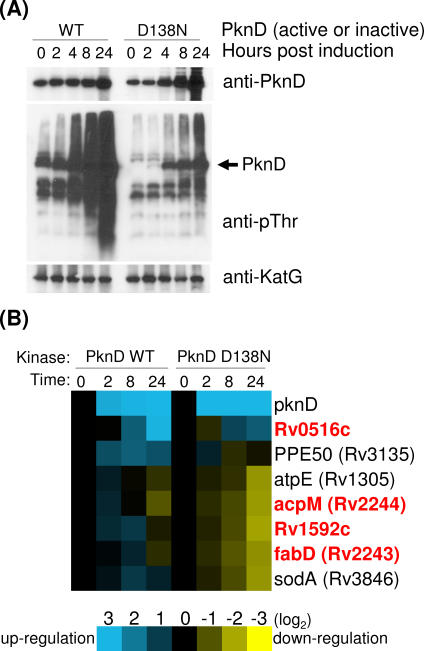
Efficient Overexpression of PknD and Concomitant Induction of Rv0516c in Logarithmically Growing *Mtb* (A) Western blots showing kinase levels (anti-PknD) and kinase activity (anti-phosphothreonine) at 0, 2, 4, 8 and 24 h after induction of WT and Asp138Asn PknD (D138N). As a loading control, the amount of the KatG protein present at a constant level was monitored using anti-KatG antibodies. Addition of the inducer (acetamide) to WT *Mtb* (lacking an inducible PknD gene) caused no discernable change in the pattern of phosphoproteins (unpublished data). The presence of a band at the molecular weight of phosphorylated WT PknD at time zero may indicate that the kinase was already expressed (and autophosphorylated) at low levels in the absence of inducer. In contrast, phosphorylated Asn138Asn PknD accumulated only after the inducer was added. (B) Cluster diagram showing the eight genes with the largest time-dependent changes in transcript levels upon PknD induction. Expression levels are shown relative to a reference pool in which equal amounts of RNA from each sample were mixed and the value at time zero was normalized to 1.0. Microarray analysis revealed >2-fold induction or repression of 137 genes (Table S1). Among the eight most altered transcripts shown here, we observed robust induction (blue) of the putative anti-anti–sigma factor Rv0516c. Rv0516c expression increased much more upon expression of WT PknD than the attenuated mutant (Asp138Asn). Genes labeled in red are among those most reduced in expression during logarithmic phase growth of an *Mtb sigF* knockout mutant [21]. Color unit is fold change of gene expression.

To investigate the pathways regulated by receptor STPK signaling, we constructed *Mtb* strains expressing either wild-type (WT) or kinase-dead (Asp138Asn) PknD under the control of an acetamide-inducible promoter [20]. The Asp138Asn mutation in the catalytic site reduced the in vitro activity of kinase domain ∼2,600-fold (Figure S1). In this approach, excess kinase substituted for an activating signal to stimulate downstream pathways. Western blotting with anti-PknD and anti-pThr antibodies showed that the WT or mutant kinases accumulated after induction and produced a concomitant increase in Thr phosphorylation (Figure 1A). Expression of the attenuated Asp138Asn mutant produced a much smaller increase in phosphorylation of cellular targets. Consistent with the idea that the expressed PknD (directly or indirectly) mediated the observed phosphorylation in vivo, cellular–protein phosphorylation was blocked when the PknD variants were induced in the presence of SP600125 (Figure S1B), a c-Jun N-terminal kinase (JNK) inhibitor that shows specificity for PknD over other *Mtb* STPKs (C. Mieczkowski and T. Alber, unpublished data).

Transcriptional profiling using microarrays confirmed the induction of *PknD* transcripts and revealed a set of genes regulated by PknD overexpression in a kinase-dependent manner (Figure 1B; Table S1). Remarkably, the transcripts most differentially expressed in the strain expressing WT PknD (Figure 1B) included the genes with the largest reductions in transcription during log phase growth of an *Mtb* mutant harboring a deletion of *sigF* [21]. Moreover, the *Rv0516c* gene, which is homologous to anti-anti–sigma factors, was dramatically induced by PknD overexpression. The established role of phosphorylation in anti-anti–sigma factor regulation supported the hypothesis that PknD specifically phosphorylates Rv0516c.

**Figure 2:**
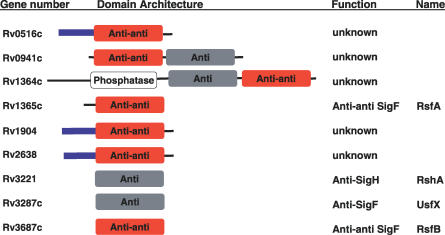
*Mtb* Proteins Containing Domains Homologous to the B. subtilis Alternative Sigma Factor Regulators SpoIIAA and SpoIIAB Four of the *Mtb* homologs of the B. subtilis SpoIIAB (anti–sigma factor) or SpoIIAA (anti-anti–sigma factor) encode proteins that have been shown to regulate SigF or SigH. *Rv0941c* contains both types of domain, and *Rv1364c* contains both domains as well as an RsbU-like, Ser/Thr phosphatase domain. Three of the seven anti-anti–sigma factor domains are preceded by a Ser/Thrrich N-terminal extension (blue). This extension in Rv0516c contains the site of PknD phosphorylation.

**Figure 3:**
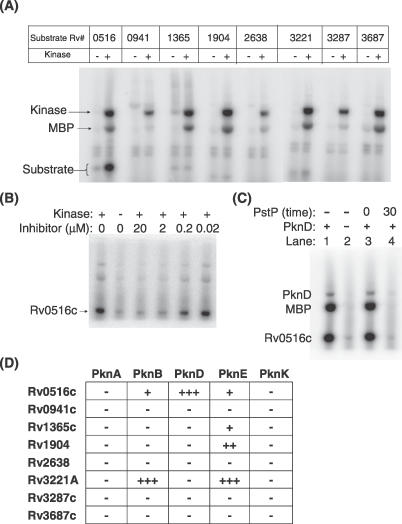
PknD Specifically Phosphorylates Rv0516c In Vitro (A) Each anti–sigma factor or anti-anti–sigma factor (gene [Rv] number listed along the top) fused to MBP was cloned, expressed, purified, and incubated with (+) or without (−) PknD in a [γ-^32^P]ATP transfer assay. The assay was quenched with EDTA, and TEV protease was added to separate the MBP tag from the sigma factor regulator. The two dark bands in the center of each PknD lane correspond to the autophosphorylated kinase and the MBP tag. The sigma factor regulators released by TEV protease are between 12 and 20 kDa. Only Rv0516c was phosphorylated by PknD. (B) Dose-dependent attenuation by the PknD inhibitor, SP600125, shows that PknD phosphorylates Rv0516c. (C) PstP, a protein Ser/Thr phosphatase from *Mtb,* dephosphorylates Rv0516c. PknD (lane 1), but not heat-inactivated PknD (lane 2), phosphorylated Rv0516c. PstP was added to PknD-phosphorylated Rv0516c, and the phosphatase reaction was quenched immediately (lane 3) or after 30 min (lane 4). (D) In vitro kinase assays were performed to investigate the specificity of five *Mtb* kinases for the eight sigma factor regulators (Figure S3). Assays were carried out in a manner identical to the assay shown in Figure 3A. Kinase concentrations were adjusted to catalyze equal phosphorylation of myelin basic protein in vitro. PknB, PknD, and PknE phosphorylated a specific subset of substrates.

To test this idea, we measured the phosphorylation by the purified PknD kinase domain of all predicted *Mtb* homologs of the B. subtilis alternative sigma factor regulators. Potential regulators were identified using iterative PSI-BLAST searches for homologs of Rv0516c, SpoIIAA, and SpoIIAB, and putative homologs were confirmed using 3D-PSSM [22] to verify that the predicted fold resembled anti– or anti-anti–sigma factors (Figure 2). All of the identified sigma factor regulators were cloned, expressed in *Escherichia coli,* and purified. Using a [γ-^32^P]ATP transfer assay, we found that the PknD kinase domain (PknD_1–378_) efficiently phosphorylated Rv0516c, but not any of the other sigma factor regulator homologs (Figure 3A). The SP600125 inhibitor blocked this Rv0516c phosphorylation (Figure 3B) in a dose-dependent manner, indicating that PknD catalyzed the observed phosphorylation. Rv0516c phosphorylation was reversed by PstP (Figure 3C), the *Mtb* protein Ser/Thr phosphatase that dephosphorylates all *Mtb* STPK substrates tested to date [23]. These results showed that PknD and PstP act on the putative anti-anti–sigma factor Rv0516c in vitro.

To determine if *Mtb* UsfX (Rv3287c), the anti–sigma factor Ser kinase that controls SigF [24], also phosphorylates Rv0516c, we incubated these proteins under conditions in which UsfX phosphorylated the model substrate, myelin basic protein (MyBP). In contrast to PknD, UsfX failed to phosphorylate Rv0516c (Figure S2). The anti–sigma factor paralogs RshA [25] and Rv0941c also failed to phosphorylate Rv0516c (unpublished data). Thus, unlike anti-anti–sigma factors that are phosphorylated by anti–sigma factors, Rv0516c is phosphorylated by a eukaryotic-like STPK, PknD.

To determine if sigma factor regulator phosphorylation is a general function of *Mtb* STPKs, we assayed the ability of four other *Mtb* kinase domains (PknA, PknB, PknE, and PknK) to phosphorylate Rv0516c and the eight other purified *Mtb* sigma factor regulators. At concentrations of each kinase domain equally active in phosphorylating the nonspecific substrate, MyBP, Rv0516c was phosphorylated most efficiently by PknD, and to a lesser extent by PknB and PknE (Figures 3D and S3). Neither PknA nor PknK phosphorylated any of the sigma factor regulators in vitro. PknB and PknE also phosphorylated the anti–sigma factor RshA (Rv3221A). The role of this phosphorylation remains to be determined, as the phosphorylation of an anti–sigma factor has not been described previously. PknE weakly phosphorylated Rv1904 and RsfA (Rv1365c). The five kinase domains tested failed to phosphorylate any of the other sigma factor regulators. The specific in vitro phosphorylation of Rv0516c by the PknD kinase domain correlated with the transcriptional stimulation of Rv0516c by PknD in vivo.

**Figure 4:**
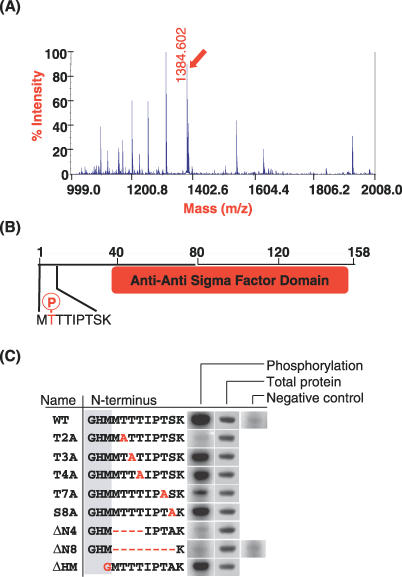
PknD Phosphorylates Rv0516c on Thr2 In Vitro (A) Mass spectrometry of trypsin-digested, PknD-phosphorylated Rv0516c indicated the presence of a single phosphorylation site within the N-terminal nine residues. The figure shows the mass spectrum of an HPLC fraction of the digest. The measured mass (monoisotopic MH^+^) of the N-terminal peptide (1384.60 Da [arrow]) was 79.97 Da larger than the expected mass of 1,304.63 Da, corresponding to addition of a single phosphate group. Tandem MS and N-terminal sequencing of the peptide at m/z 1,384.60 revealed Thr2 as the site of phosphorylation. (B) Rv0516c Thr2 occurs in a unique, 28-residue Ser/Thr-rich, N-terminal extension, not in the anti-anti–sigma factor domain. (C) Mutations in the N-terminus of Rv0516c confirm that PknD phosphorylates Rv0516c on Thr2. The eight indicated mutants of Rv0516c were purified, and in vitro phosphorylation by the PknD kinase domain was assayed after cleavage with the TEV protease (left). Non-native residues before the start of Rv0516c left after TEV protease cleavage are shaded in gray. Protein-stained loading controls (center) confirmed that an equal amount of each Rv0516c variant was assayed. Controls lacking kinase showed that the phosphorylation was due to PknD (far right). Thr2Ala (T2A) and deletions of the N-terminal four and eight residues caused the most dramatic reductions in phosphorylation.

To explore the mechanism of PknD regulation of Rv0516c, we determined the site of Rv0516c phosphorylation by mass spectrometry and protein sequencing (Figure 4). Electrospray ion-trap mass spectrometry revealed a molecular mass of 17,392.4 ± 1 for purified, recombinant Rv0516c phosphorylated to completion in vitro using the PknD kinase domain. This mass corresponded to mono-phosphorylated Rv0516c (the expected M_R_ of the unphosphorylated protein is 17,312.7). Matrix-assisted laser desorption ionization (MALDI) tandem time-of-flight (TOF) analysis of trypsin-digested phospho-Rv0516c indicated that the peptide consisting of the N-terminal nine residues was the only phosphopeptide (Figure 4A). Tandem MS and N-terminal sequencing showed that Thr2 accounted for all of the Rv0516c phosphorylation (Figure 4B). Eight Rv0516c N-terminal mutants—individual Ala substitutions of the five Ser or Thr residues in the N-terminal segment as well as deletions of up to eight residues—were created to confirm phosphorylation at Thr2 (Figure 4C). As expected, PknD failed to phosphorylate the Thr2Ala Rv0516c and the two deletion mutants lacking Thr2. The Thr7Ala variant showed a reduction in phosphorylation, suggesting that Thr7 plays a role in kinase binding and recognition. These data demonstrated that PknD phosphorylates Rv0516c on a unique N-terminal site, Thr2.

**Figure 5:**
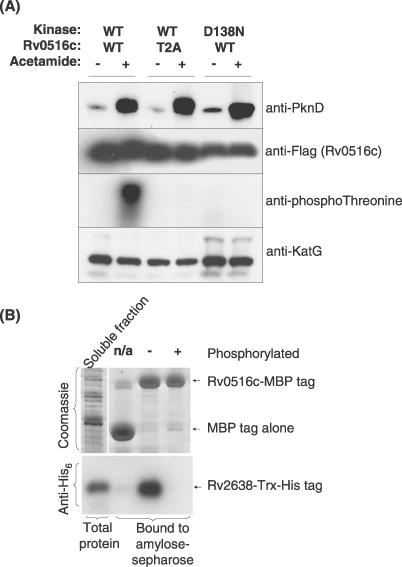
PknD Phosphorylates Rv0516c on Thr2 In Vivo, and This Phosphorylation Blocks Binding to Another Sigma Factor Regulator (A) Western blot of lysates of *Mtb* strains expressing full-length PknD under the control of an acetamide-inducible promoter and constitutively expressing Rv0516c with a C-terminal, FLAG epitope tag. As controls, the kinase was rendered inactive (D138N) or the Rv0516c phosphorylation site was mutated (Thr2Ala [T2A]). The active (but not the mutant) PknD phosphorylated the overexpressed WT Rv0516c. The levels of endogenous proteins were too low to detect phosphorylated Rv0516c in this experiment. KatG was detected with antibodies as a loading control. Rv0516c (but not the Thr2Ala mutant) was phosphorylated upon WT PknD induction. (B) Phosphorylation on Thr2 abolished binding to Rv2638. Equal amounts of cell lysates overexpressing Rv2638 were incubated with pre-phosphorylated (lane 4) or unphosphorylated (lane 3) Rv0516c. As a control, Rv2638 binding to the purification tag alone was evaluated (lane 2). Protein staining was used to insure that equal amounts of recombinant protein were used as bait in each reaction, and bound Rv2638 was visualized by Western blotting with antibodies against the His_6_ tag. Total soluble protein before incubation with the amylose-Sepharose (lane 1) indicated the presence of Rv2638. Only unphosphorylated Rv0516c bound the anti-anti–sigma factor, Rv2638.

To determine if PknD phosphorylates Rv0516c at Thr2 in vivo, we constructed *Mtb* strains that co-expressed Rv0516c (WT or Thr2Ala fused to a C-terminal 3XFLAG tag and expressed using a GroEL promoter) and full-length PknD (WT or kinase-dead with no tag expressed using an acetamide-inducible promoter). Western blots confirmed equivalent Rv0516c and PknD overexpression in each strain (Figure 5A). Using anti–phospho-Thr antibodies, we found that Rv0516c was efficiently phosphorylated only in the strain overexpressing WT PknD and WT Rv0516c. Mutations that inhibited PknD (Asp138Asn) or eliminated the Rv0516c in vitro phosphorylation site (Thr2Ala) abolished this phosphorylation of recombinant Rv0516c in *Mtb*. These data indicated that PknD phosphorylated Rv0516c on Thr2 in vivo when both proteins were overexpressed.

Although some anti-anti–sigma factor homologs of Rv0516c are known to be regulated by phosphorylation, Thr2 differs from the consensus phosphorylation sites in the anti-anti–sigma factor family. In Rv0516c, Gly80 occupies the position of the consensus Ser or Thr phosphorylation site, which occurs, for example, at Ser58 of SpoIIAA (Figure S4A). This consensus Ser/Thr phosphorylation site is conserved in only two of the six *Mtb* anti-anti–sigma factor domains (Figure S4B), but oxidation of cysteine at this position plays a key regulatory role for at least Rv1365c [24]. The Rv0516c peptide containing the corresponding segment was identified exclusively in an unphosphorylated form (unpublished data) in our MS analysis of PknD-phosphorylated Rv0516c. The absence of a Ser or Thr at the consensus phosphorylation site and our failure to observe Rv0516c phosphorylation by any anti–sigma factor in vitro are consistent with PknD phosphorylation at the distinct site, Thr2.

A yeast two-hybrid analysis of interactions among *Mtb* sigma factor regulators has suggested that Rv0516c can bind the homologous predicted anti-anti–sigma factor, Rv2638 [18]. To test this association and investigate the role of Thr2 phosphorylation in regulating the interaction, we used affinity chromatography to compare binding of purified Rv2638 to Rv0516c before and after PknD phosphorylation. Rv2638 bound Rv0516c, and Rv0516c phosphorylation by PknD abolished this association (Figure 5B). These results showed that Thr2 phosphorylation regulates the interaction in vitro between Rv0516c and the anti-anti–sigma factor paralog, Rv2638.

## Discussion

Because the identity of activating environmental signals remains unknown, we stimulated PknD receptor kinase activity in *Mtb* by overexpressing the protein (Figure 1A). Overexpression was expected to stimulate phosphorylation of PknD substrates directly by increasing the concentration of the kinase and indirectly by favoring dimerization (by mass action), which leads to allosteric activation [26]. PknD activity produced a transcriptional response that altered genes activated by SigF during log phase growth (Figure 1B), including the anti-anti–sigma factor homolog Rv0516c [21]. Strikingly, the PknD kinase domain also directly phosphorylated the Rv0516c protein (but none of the other *Mtb* sigma factor regulators) in vitro and upon overexpression in vivo. In contrast to the conserved internal phosphorylation sites found in many anti-anti–sigma factors [16], PknD phosphorylated Rv0516c on Thr2 in an N-terminal extension similar to that found in two additional mycobacterial anti-anti–sigma factors, Rv1904 and Rv2638. Phosphorylation directly blocked Rv0516c binding to Rv2638, indicating that Thr2 phosphorylation has a direct functional consequence. Although the roles of the Rv0516c:Rv2638 complex are unknown, alternative phosphorylation sites and functional interactions between upstream anti-anti–sigma factor homologs have been demonstrated in B. subtilis for the RsbS and RsbRA–RsbRD regulators [17,27–29]. These B. subtilis regulators form a large complex that controls the environmental sensing phosphatase RsbU, which dephosphorylates anti-anti–sigma factors [17,27–29].

The correlation between SigF-responsive genes [21] and genes that are transcriptionally sensitive to PknD activity (Figure 1) is specific to PknD; overexpression of *Mtb* PknB produces a distinct transcriptional response (T. Lombana, J. MacGurn, J. Cox, and T. Alber, unpublished data). Nonetheless, these data do not demonstrate a direct mechanistic link between Rv0516c and SigF. To the contrary, the lack of Rv0516c phosphorylation by the *Mtb* anti-SigF (UsfX, Rv3287c) or any other anti–sigma factor hints that PknD indirectly influences SigF-mediated transcription by phosphorylating Rv0516c or other substrates. The present data do not distinguish models in which repression of the SigF response is caused by Rv0516c phosphorylation or by a distinct signal generated by phosphorylation of one or more other proteins in vivo.

The substrates of the *Mtb* STPKs are not restricted to transcriptional regulators. Rough estimates suggest that the number of Ser/Thr phosphoproteins in *Mtb* may exceed 100 [30], and proposed *Mtb* substrates include metabolic enzymes [31], regulatory proteins [12,32], and membrane channels [33]. Using proteomic methods to analyze lysates phosphorylated in vitro by the PknD kinase domain, Perez and coworkers recently reported that PknD phosphorylates MmpL7, the transporter for phthiocerol dimycocerosate (PDIM), a lipid essential for virulence [34]. These studies, however, did not test whether MmpL7 is phosphorylated in vivo, whether phosphorylation altered the function of MmpL7, or whether MmpL7 is a better substrate of other STPKs. Perez and coworkers did not detect PknD phosphorylation of Rv0516c, perhaps because this regulatory protein may not be sufficiently abundant in the *Mtb* lysates or because proteins <20 kDa (such as Rv0516c) were run off the two-dimensional gels used to identify potential substrates [34]. Similar reasons may explain the failure of Perez and coworkers to detect phosphorylation of small proteins containing FHA domains previously found to be in vitro substrates of PknD [9].

In contrast, the complementary approach used here, based on assaying the biochemical and transcriptional effects of kinase activation in vivo, is sensitive to changes in the activity of regulatory factors, even proteins present in small amounts. By assaying in vitro PknD phosphorylation of all the *Mtb* homologs of the B. subtilis SpoIIAA and SpoIIAB sigma factor regulators, we found that only Rv0516c was efficiently phosphorylated (Figure 3). Nonetheless, overexpressing PknD may cause abnormal phosphorylation or physiological changes that result in indirect transcriptional changes unrelated to normal kinase functions. The striking correlation between PknD phosphorylation of Rv0516c and activation of the *Rv0516c* gene, however, suggests a potential autoregulatory loop and sets the stage to explore the biological roles of this sigma factor regulator and the consequences of Rv0516c phosphorylation in vivo.

Although bacterial STPKs phosphorylate many types of proteins [30], alternative sigma factor regulators may be substrates of STPKs in diverse genera. In addition to the activity of PknD, the PknB and PknE kinase domains phosphorylated sigma factor regulators in vitro (Figures 3D and S3). In contrast, some kinase domains (e.g., PknA and PknK; Figure 3D) apparently do not phosphorylate these sigma factor regulators. With up to 12 candidate alternative sigma factors in the *Mtb* genome, it is unlikely that each kinase controls a completely autonomous pathway. Instead, our data suggest that phosphorylation pathways may converge on overlapping sets of regulators (Figure 3D). The specific phosphorylation of Rv0516c on a novel functional site by PknD suggests that STPK phosphorylation of sigma factor regulators goes beyond the paradigm established to date in B. subtilis.

## Materials and Methods

### Bacterial strains, media, and growth conditions.

The strains and plasmids used in this study are listed in Table 1. M. tuberculosis (Erdman) cultures were grown in 7H9 medium and transformed as previously described [35]. Plasmids were maintained episomally by growth in medium containing antibiotics.

**Table 1:**
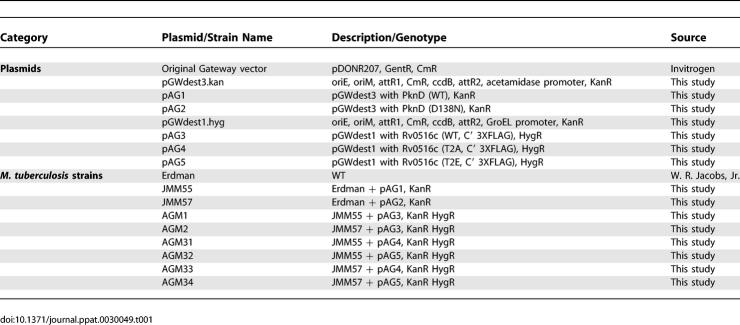
Plasmids and Strains Used in This Study

### Microarrays: Data collection and analysis.

Strains were grown to mid-log phase in 7H9 media before induction of PknD by addition of acetamide (0.2%). RNA was isolated from cultures at indicated time points as previously described [36] and quantified by measuring OD_260_. RNA was random-primed and reverse transcribed in the presence of amino-allyl dUTP. Residual RNA was hydrolyzed by addition of 0.2 N NaOH, 0.1 M EDTA, and incubation at 65 °C for 15 min, followed by addition of 0.2 N HCl to neutralize. The cDNA was purified with Zymo binding columns (Zymo Research, http://www.zymoresearch.com) and conjugated to either Cy3 (individual cDNA samples) or Cy5 (common reference pool cDNA). An equal quantity of each RNA sample within an experiment (representing both *Mtb* strains at all time points) was used to make a common cDNA reference pool. Dye-conjugated cDNA from each individual sample was mixed and co-hybridized with dye-conjugated cDNA from the common reference pool on microarray slides containing oligonucleotide spots representing every gene in M. tuberculosis (Qiagen, http://www.qiagen.com). After 2 d of hybridization at 63 °C, arrays were washed and scanned using a GenePix 3000B scanner (Axon Instruments, http://www.moleculardevices.com). Array gridding was performed in GenePix Pro 4.1, and Nomad 2.0 was used to select high quality spots. For each spot, the ratio of medians (Rm) was averaged from repeat hybridizations and normalized to *t* = 0 (uninduced). Cluster analysis was performed using Cluster 3.0. Two biological replicates were performed, and each biological replicate was averaged over two hybridizations.

### Protein expression and purification for in vitro assays.

Using *Mtb* H37Rv genomic DNA as a template for PCR amplification, gene segments encoding PknB_1–308_, PknE_1–286_, and PknK_1–289_ were cloned into pET-28b vectors (Novagen, http://www.emdbiosciences.com). PknD_1–378_ was cloned into pET-24b (Novagen). PknA_1–337_ and full-length clones of each anti–sigma factor or anti-anti–sigma factor were inserted into the Gateway vector pHMGWA [37], which included NH_2_-terminal 6X-His and maltose binding protein (MBP) tags, followed by a tobacco etch virus (TEV) protease site. All constructs were confirmed by DNA sequencing.

Proteins were expressed in E. coli BL21 CodonPlus (Stratagene, http://www.stratagene.com) at 18 °C. The kinase-domain constructs and Rv0516c were purified to homogeneity (as assayed by SDS-PAGE) by immobilized metal affinity chromatography (IMAC) using nickel-equilibrated HiTrap chelating Sepharose (Amersham, http://www.amershambiosciences.com), size-exclusion chromatography using HiLoad 26/60 Superdex 75 (Amersham), and anion-exchange chromatography using HiTrap Q Sepharose (Amersham). The sigma factor regulators prepared for kinase-activity screens were purified by nickel-IMAC (Rv0516c was purified by IMAC only for these assays as well). The molecular weight of each sigma factor regulator, as assayed by SDS-PAGE, corresponded to the mass predicted by the gene sequence. Because the kinase-domain constructs autophosphorylated during expression, migration on SDS-PAGE was slightly retarded.

### In vitro kinase assays.

The sigma factor regulators were dialyzed into the reaction buffer (80 mM NaCl, 20 mM Tris [pH 7.5], 0.5 mM Tris(2-carboxyethyl)phosphine hydrochloride [TCEP], 250 μM MnCl_2_). In a total reaction volume of 19 μl, the final concentration of each IMAC-purified 6X-His-MBP–tagged sigma factor regulator was 20 μM, and the final concentration of kinase was 1.2 μM. The reaction was initiated with the simultaneous addition of 1 μL of [γ-^32^P]ATP (800 Ci/mmol and 10 mCi/ml; ICN, http://www.mpbio.com) and ATP (Sigma, http://www.sigmaaldrich.com) to final concentrations of 250 nCi/μl and 50 μM, respectively. The reaction was allowed to proceed for 2 h at room temperature and quenched by the simultaneous addition of EDTA to 20 mM and 7.2 μg of TEV protease. The TEV cleavage was allowed to proceed 2 h or overnight at room temperature, resulting in efficient separation of each sigma factor regulator from the tag. The sequence GlyHisMet was left at the NH_2_-terminus after TEV cleavage of the tag. The cleavage reactions were separated by SDS-PAGE on 4%–12% NuPage Novex BisTris gels (Invitrogen, http://www.invitrogen.com), and the gels were dried. Radioactivity was quantified with a Molecular Dynamics Typhoon 8600 phosphoimager.

### Activity of the Asp138Asn mutant.

To assess the activity of the Asp138Asn mutant of PknD, we incubated 0.036 nM WT kinase or 3.6 nM Asp138Asn kinase with 0.5 mg/ml MyBP or 0.5 mg/ml Rv0516c. The reaction was carried out for 30 min under buffer, metal, and ATP concentrations similar to those described above, and then quenched with either 5X SDS-PAGE loading dye (for MyBP) or the TEV/EDTA mixture described above. Phosphorylation was quantified with ImageQuant (GE Healthcare, http://www.gehealthcare.com) after electrophoresis, drying, and phosphoimager data collection.

### PknD inhibition by SP600125.

Untagged PknD_1–378_ was purified by IMAC, cleaved with TEV, purified on HiLoad 26/60 Superdex 75 (Amersham), and concentrated from the flow-through fraction of a second IMAC column. Each reaction was set up with or without 38 nM kinase, 20 μM Rv0516c, 250 nCi/μl [γ-^32^P]ATP, and 25 μM unlabeled ATP in 50 mM NaCl, 50 mM HEPES (pH 7.5), 0.5 mM TCEP, 10 mM MnCl_2_, and 10 mM MgCl_2_. SP600125 was diluted into water and added to a concentration of 20 nM to 20 μM. Reactions were carried out and analyzed as described above.

### In vitro dephosphorylation of Rv0516c by PstP.

PknD, Rv0516c, and PstP were purified to homogeneity [30]. Heat-inactivated PknD was prepared by incubation at 95 °C for 1 h. Phospho-Rv0516c prepared in a 2-h incubation with PknD and [γ-^32^P]ATP was treated with 2.3 μg of PstP. The reaction was quenched with EDTA and TEV after zero or 30 min. Separation and quantification were carried out as described above.

### Phosphorylation site mapping.

Purified Rv0516c was phosphorylated using 6X-His-PknD_1–378_ and 2 mM ATP. The reaction proceeded overnight, and the kinase was removed by IMAC. The flow-through fraction was diluted with water and rocked at room temperature for 2 d to induce precipitation. Supernatant was removed, and the resulting pellet was dissolved in 6 M guanidinium hydrochloride.

The mass of the intact protein was determined by electrospray ionization–ion-trap mass spectrometry. Rv0516c was digested with trypsin; the resulting digest mixture was separated on a reversed-phase C-18 column (0.15 × 150 mm), and fractions were collected. The MALDI TOF spectrum of each fraction was obtained, and the phosphorylated peptide was identified using MALDI-tandem TOF (MS/MS). The MS/MS spectrum was used, along with Edman sequencing, to identify the phosphorylation site.

Mutations to the N-terminus of Rv0516c were created with QuikChange (Stratagene). Proteins were purified and phosphorylated as described, except that the Rv0516c variants were treated with TEV protease and quenched with the protease inhibitor, aminoethyl-benzene sulfonyl fluoride (AEBSF) (MP Biomedicals, http://www.mpbio.com), prior to phosphorylation.

### In vivo phosphorylation state analysis.

Full-length PknD was cloned into an acetamide-inducible M. tuberculosis expression vector (pGWdest3.kan) [38]. Full-length Rv0516c (WT or mutant) was cloned into a tuberculosis expression vector under the control of the constitutive GroEL promoter (pGWdest1.hyg). The vector also encoded a C-terminal antigenic FLAG (DDDDK) tag. Mutants were made using Quikchange (Stratagene) on the *Rv0516c* gene in the entry vector. *Mtb* Erdman was transformed by electroporation [35], grown for 3–6 wk on solid rich medium, and single colonies were picked and grown to mid-log phase. Large (50 mL) cultures were inoculated and adjusted to an optical density (OD) at 600 nm of 0.3 after 5 d. To induce PknD expression, acetamide was added to a final concentration of 0.2% at 24, 8, 4, or 2 h before harvesting. Induced and uninduced cultures were grown to a final OD_600_ of ∼0.6. Then, 10 mL of each culture were harvested by centrifugation, and resuspended in 200 μL of extraction buffer (1% SDS, 20 mM EDTA, 50 mM HEPES (pH 7.5), 1 mM AEBSF). Samples were immediately boiled for 25 min. Next, 200 μL of 100-μm glass beads were added, and samples were bead-beaten twice for 5 min. Samples were boiled a second time for 10 min and centrifuged. The soluble fraction was removed and diluted with SDS-PAGE loading dye. After electrophoresis in 10%–20% Tris-glycine gels, proteins were transferred to PVDF membrane, and detected with anti-phosphoThreonine (Invitrogen), anti-DDDDK (AbCam), anti-KatG, or anti-PknD antibodies (Pacific Immunology, http://www.pacificimmunology.com). HRP-conjugated secondary antibody was used with Kodak BioMax MR film to develop the Western blots. In cases of multiple antibody detection, blots were stripped in 2% SDS/100 mM DTT, 62 mM Tris (pH 7), for 30 min at 50 °C.

### In vitro interaction assays.

Rv2638 was expressed in E. coli in the pHxGWA (N-terminal His_6_-thioredoxin) vector [37]. Phosphorylated Rv0516c was prepared by incubation of purified His_6_-MBP–tagged protein with 5 mM MnCl_2_, 2 mM ATP, and 10:1 (w:w) PknD_1–378_. Phospho-Rv0516c was purified by IMAC, and both phosphorylated and unphosphorylated Rv0516c were dialyzed into the pull-down buffer (70 mM NaCl, 20 mM HEPES 7.5, 0.5 mM TCEP).

Pull-downs were performed by lysing (by sonication) E. coli that expressed Rv2638 in the presence of 200 μg MBP-tagged Rv0516c (phosphorylated or unphosphorylated) or His_6_-MBP control in 70 mM NaCl, 20 mM HEPES 7.5, 0.5 mM TCEP, 1 mM AEBSF, and 10 mM MnCl_2_. After rocking the lysate for 30 min at 4 °C, samples were centrifuged for 10 min at 14,000 rpm in a microcentrifuge at 4 °C. A small amount of the supernatant was retained for analysis, and the majority was applied to 50 μL of amylose-Sepharose (New England Biolabs, http://www.neb.com) pre-equilibrated in the buffer. After rocking for 10 min at 4 °C, resin was washed three times in buffer, resuspended in 40 μL of 2X SDS-PAGE loading dye, and boiled for 10 min. Samples were separated on 12% Tris-glycine gels (Invitrogen), transferred to PVDF, blocked in 4% non-fat dry milk, and incubated overnight at room temperature with 1:2000 monoclonal anti-His_6_ clone HIS-1 (Sigma). Blots were washed and imaged as described above. For loading controls, the same reactions were separated on 12% Tris-glycine gels and stained with Coomassie blue.

**Table 2:**
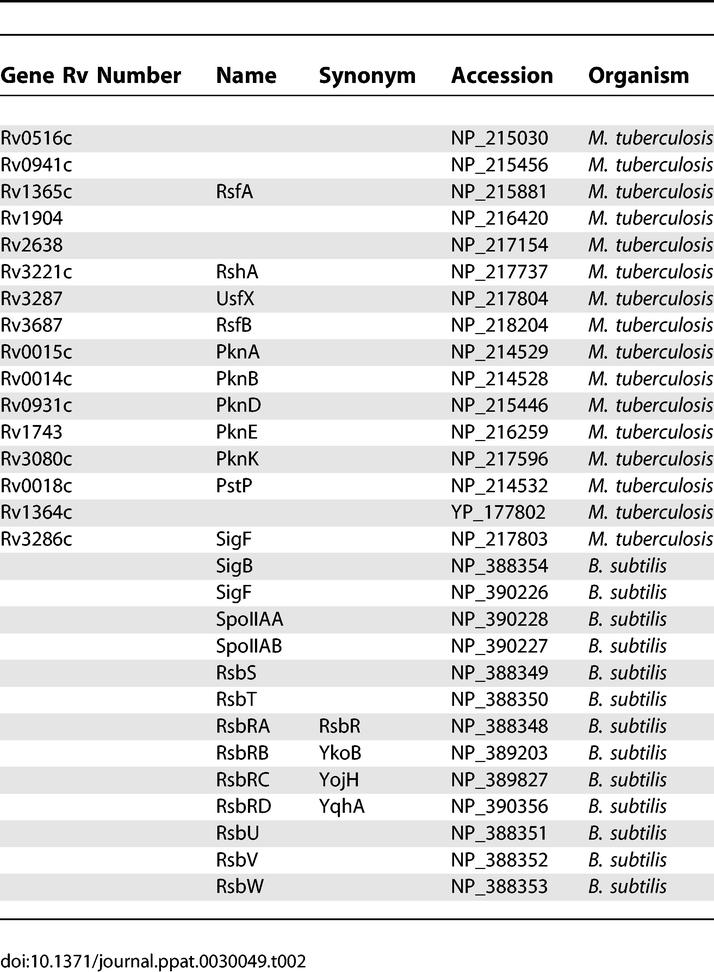
GenBank Accession Numbers of Genes Described in This Study

## Supporting Information

Figure S1 Inhibition of PknD (A) The D138N mutant of PknD is 2,600-fold less active than WT in vitro. Using a 100-fold excess of PknD D138N, we measured a 27.2-fold difference in Rv0516c phosphorylation, and a 25.3-fold difference in MyBP phosphorylation compared to the WT PknD kinase domain. Values were averaged over three replicates.
(B) The PknD inhibitor SP600125 delays changes in phosphorylation upon PknD expression in *Mtb. Mtb* strains expressing WT (left) or D138N PknD (right) were induced with or without the simultaneous addition of 60 μM SP600125 and grown for 8 or 24 h. All samples were lysed at the same time, clarified by centrifugation, separated by SDS-PAGE, and probed with anti-pThr antibodies. The presence of the inhibitor, which is a green compound, turned the cells green, indicating that it was likely taken up. The WT PknD, but not the D138N mutant, was present and phosphorylated in the uninduced cells (compare the 0 time points). At 8 h, SP600125 inhibited cellular protein phosphorylation by the expressed PknD. The D138N mutant PknD, however, was phosphorylated at 8 h in the presence of the inhibitor. This increased phosphorylation suggests that the overexpressed PknD is phosphorylated by other cellular kinases that are not inhibited by SP600125. By 24 h, perhaps due to overexpression of PknD or efflux of the compound, *Mtb* cellular proteins are highly phosphorylated.
(239 KB PDF)

Figure S2 Rv3287c (*Mtb* UsfX) Phosphorylates the Model Substrate MyBP, but Not Rv0516c Autoradiogram showing substrate phosphorylation in the presence (+) and absence (−) of the anti–sigma factor kinase, Rv3287.
(44 KB PDF)

Figure S3 Multiple *Mtb* Ser/Thr Protein Kinases Phosphorylate a Unique Set of Putative Sigma-Regulators The phosphorylation of the putative anti– and anti-anti–sigma factors by PknA, PknB, PknE, and PknK was assessed. PknD did not phosphorylate Rv1364c (unpublished data), but this regulator was omitted from the analysis due to the presence of a phosphatase domain. Each kinase domain was incubated in assay buffer with MBP-tagged substrate protein. The tag was removed with TEV protease and the phosphoproteins were visualized by autoradiography of SDS gels. The MBP tag was phosphorylated by the PknA, PknB, and PknE kinase domains. The kinase-domain concentrations were adjusted to produce similar phosphorylation of the nonspecific substrate, MyBP. Sigma factor regulators migrated in the bottom half of each gel. (A) PknA does not phosphorylate any sigma factor regulators. (B) PknB phosphorylates Rv0516c and Rv3221A (RshA). (C) PknE phosphorylates Rv0516c, Rv1365c (RsfA), Rv1904, and Rv3221A (RshA). (D) PknK does not phosphorylate any of these sigma factor regulators.
(624 KB PDF)

Figure S4 Multiple Sequence Alignment around the Conserved Anti-Anti–Sigma Factor Phosphorylation Site Rv0516c and other putative *Mtb* anti-anti–sigma factors are highly homologous to known anti-anti–sigma factors. For example, Rv0516c and Rv2638 align to the well-characterized RsfA (anti-anti–sigma factor F, Rv1365c) with e-values of 2 × 10^−5^ and 1 × 10^−29^, respectively.
(A) A multiple sequence alignment of anti-anti–sigma factors and Rv0516c around the phosphorylation site in B. subtilis SpoIIAA (shaded). Phosphorylation of all of these anti-anti–sigma factors has been observed in the respective bacteria. While Rv0516c contains a serine adjacent to the conserved phosphorylation site, the conserved Ser/Thr is replaced with a Gly in Rv0516c.
(B) Multiple sequence alignment showing that the phosphorylation site observed in SpoIIAA and RsbV from B. subtilis is conserved only in two of the six *Mtb* anti-anti–sigma factor domains. Oxidation of the highlighted cysteine in Rv1365c has been shown to mimic the physiological role of Ser phosphorylation.
(21 KB PDF)

Table S1 Genes Differentially Regulated in Response to Overexpression of WT or Kinase-Dead PknD For each gene at each time point, the value in the table represents the (averaged) ratio of medians (Rm) which has been normalized to the Rm at *t* = 0. Rm is defined as the median pixel intensity for the reference pool divided by the median pixel intensity of the sample. Genes depicted in this table exhibit at least a 2-fold difference in Rm between WT and kinase-dead samples at any time point. The data in the table were ordered by hierarchical clustering using Gene Cluster 3.0. N.D. indicates no data available. Genes under the control of SigF during log phase growth (e.g., *acpM, Rv1592c,* and *fabD*) were expressed at a lower level at *t* = 0 in the strain harboring the WT PknD expression plasmid compared to the kinase-dead PknD plasmid. These genes showed little fluctuation in transcriptional levels as WT PknD was overexpressed for 2, 8, or 24 h. In contrast, as expression of the kinase-dead PknD increased over time, these genes under SigF control during log phase growth were progressively repressed to levels similar to those in the strain expressing WT PknD. This pattern coincided with the leaky expression of active phosphorylated WT PknD at *t* = 0, while kinase activity was stimulated only after 4 h of kinase-dead PknD overexpression (Figure 1A). The idea that active WT PknD may have been signaling in uninduced cells is supported by the finding that the average Rm value for five representative SigF regulated genes *(acpM, Rv1592c, fabD, atpE* and *sodA)* at time zero in WT versus the Asp138Asn mutant strain was 1.9. By comparison, the mean and median Rm ratios at time zero for all genes were 1.07 and 0.96, respectively. These data suggest that some effects of WT PknD overexpression may have been manifest at *t* = 0 in these microarray experiments.
(415 KB DOC)

### Accession Numbers

Please see Table 2 for a list of genes described in this study.

## Abbreviations

MBP: maltose binding protein
*Mtb,*: *Mycobacterium tuberculosis;*
MyBP: myelin basic protein
Pkn[letter]: protein kinase [letter]
SigF: sigma factor F
STPK: Ser/Thr protein kinase
TEV: tobacco etch virus
WT: wild-type
