# Supplementary material for: M. tuberculosis Ser/Thr Protein Kinase D Phosphorylates an Anti-Anti–Sigma Factor Homolog
Source: PLoS Pathog. 2007 Apr 6;3(4):e49. doi: 10.1371/journal.ppat.0030049 (PMC1847690; doi:10.1371/journal.ppat.0030049)
Supplement: Supplementary file 1 [file ppat.0030049.sg001.pdf]

Figure S1

(A)

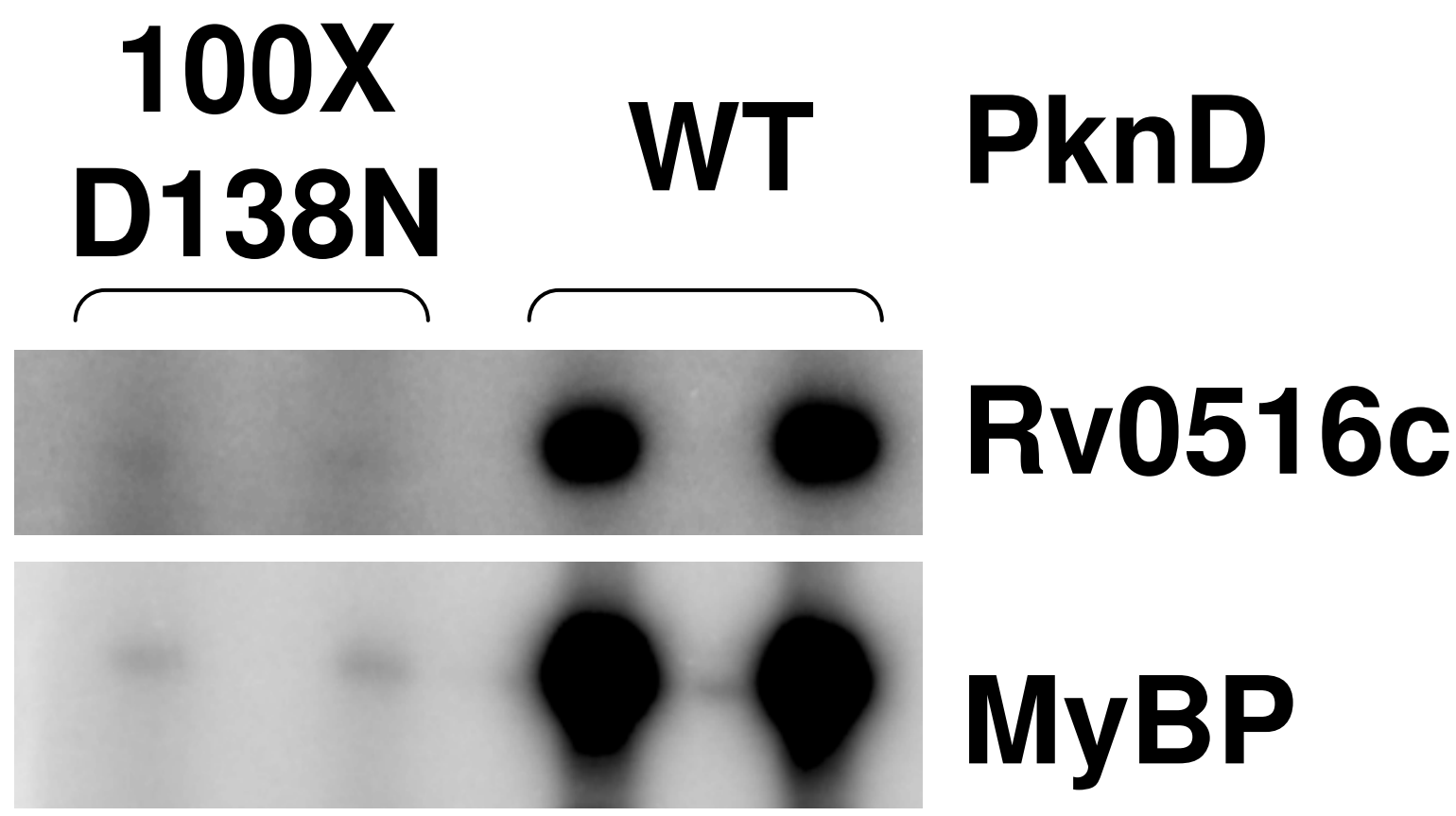
100X
D138N
WT
PknD
Rv0516c
MyBP

(B)

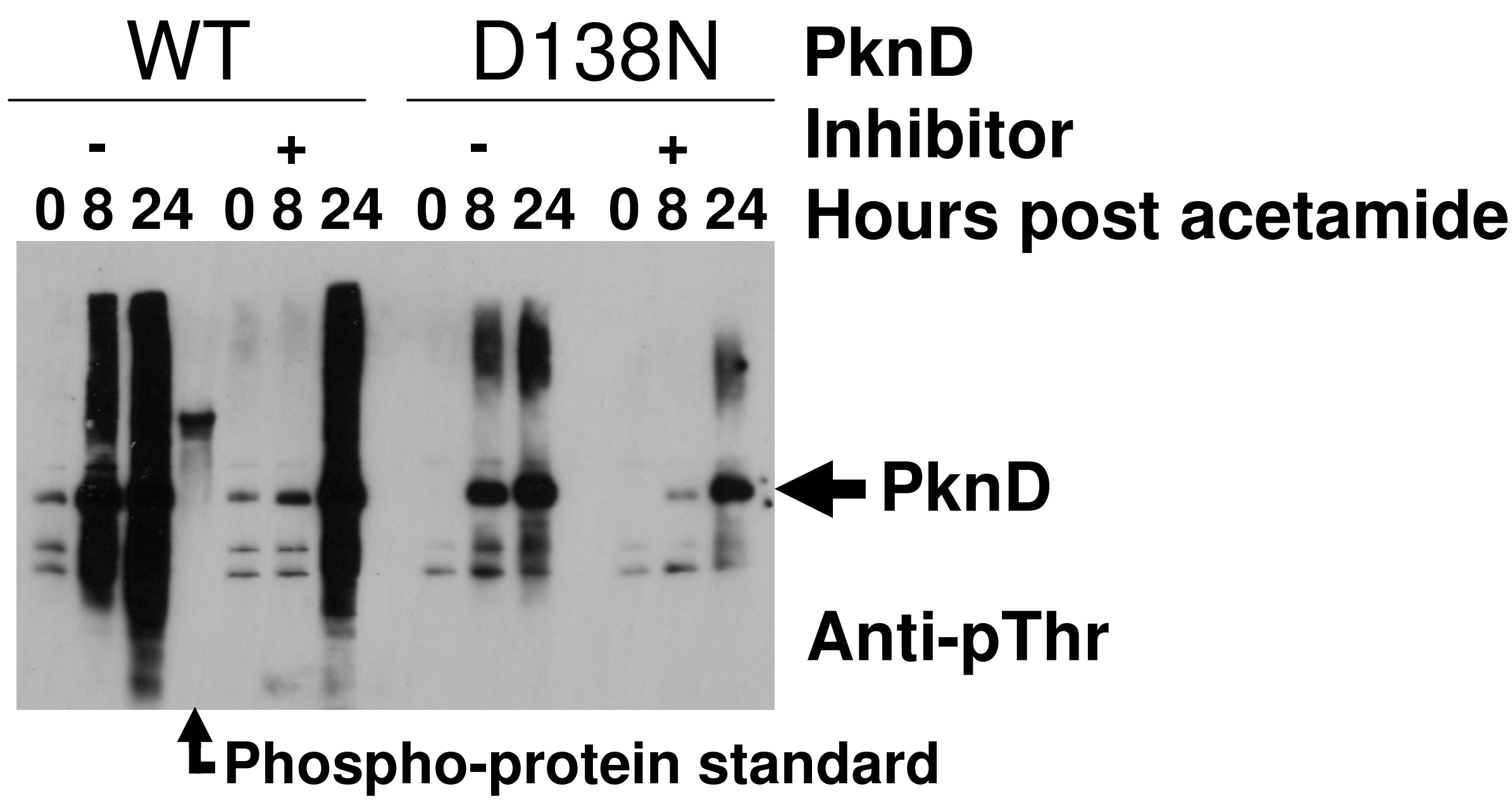
WT
D138N
PknD
Inhibitor
- + - +
0 8 24 0 8 24 0 8 24 0 8 24
Hours post acetamide
PknD
Anti-pThr
Phospho-protein standard
