# Supplementary material for: M. tuberculosis Ser/Thr Protein Kinase D Phosphorylates an Anti-Anti–Sigma Factor Homolog
Source: PLoS Pathog. 2007 Apr 6;3(4):e49. doi: 10.1371/journal.ppat.0030049 (PMC1847690; doi:10.1371/journal.ppat.0030049)

Figure S2

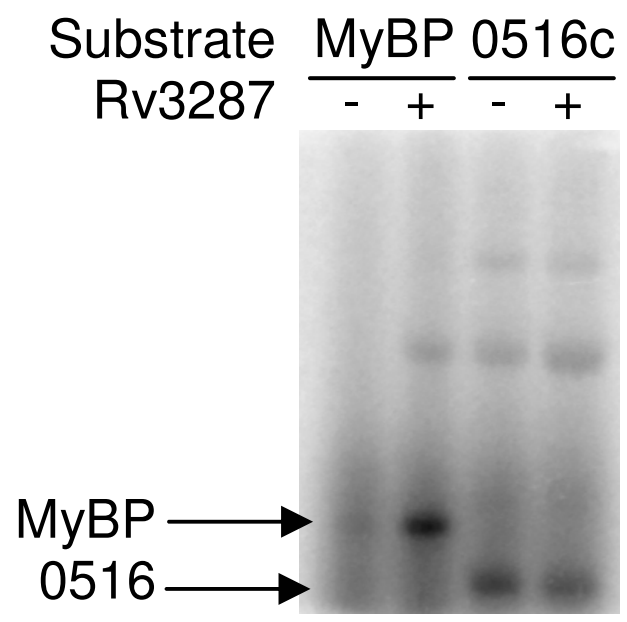

Supplement: Supplementary file 2 [file ppat.0030049.sg002.pdf]
