# Supplementary material for: M. tuberculosis Ser/Thr Protein Kinase D Phosphorylates an Anti-Anti–Sigma Factor Homolog
Source: PLoS Pathog. 2007 Apr 6;3(4):e49. doi: 10.1371/journal.ppat.0030049 (PMC1847690; doi:10.1371/journal.ppat.0030049)
Supplement: Supplementary file 3 [file ppat.0030049.sg003.pdf]

Figure S3

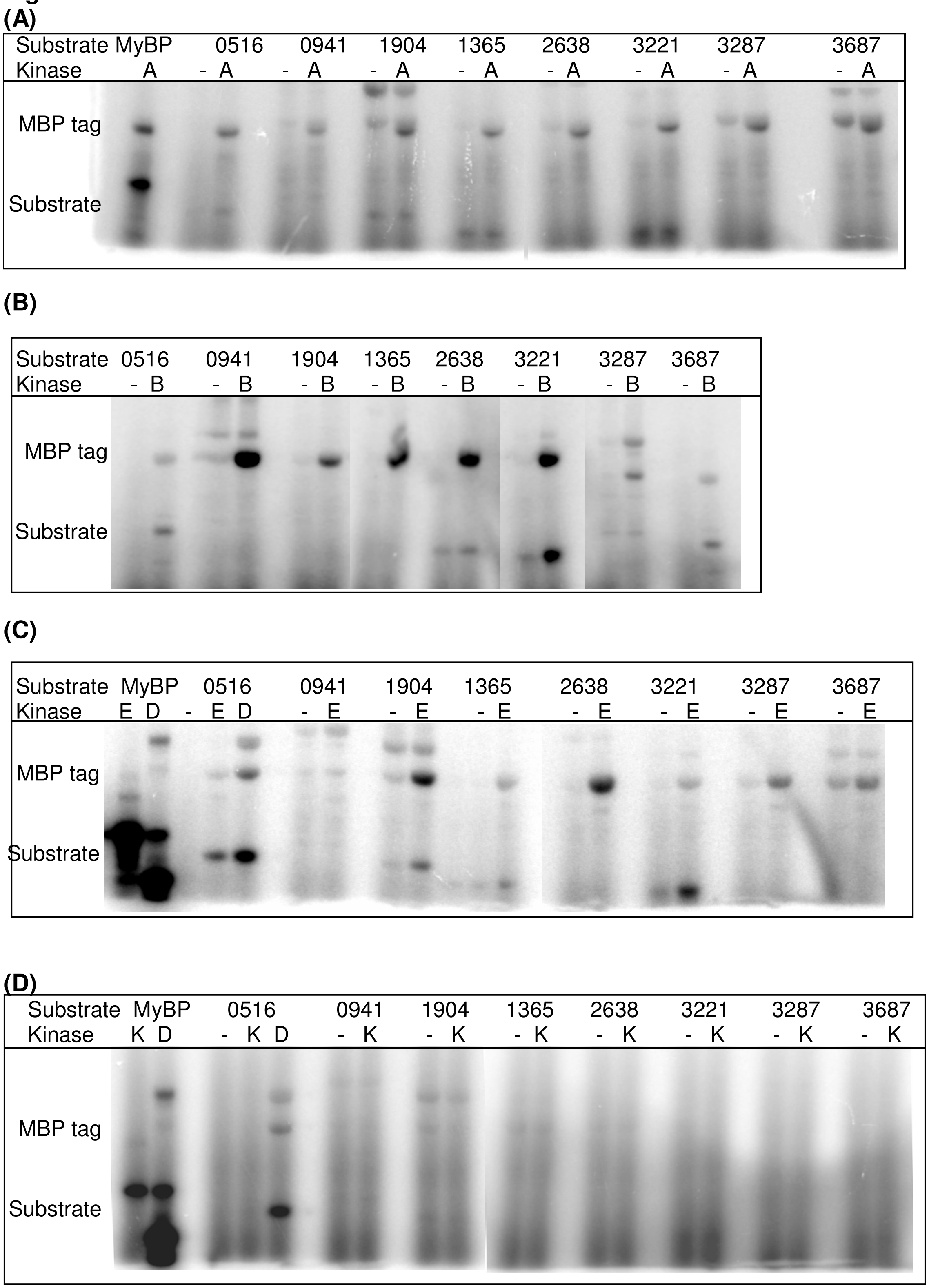
(A)
Substrate MyBP 0516 0941 1904 1365 2638 3221 3287 3687
Kinase A - A - A - A - A - A - A - A - A
MBP tag
Substrate
(B)
Substrate 0516 0941 1904 1365 2638 3221 3287 3687
Kinase - B - B - B - B - B - B - B - B
MBP tag
Substrate
(C)
Substrate MyBP 0516 0941 1904 1365 2638 3221 3287 3687
Kinase E D - E D - E - E - E - E - E - E - E
MBP tag
Substrate
(D)
Substrate MyBP 0516 0941 1904 1365 2638 3221 3287 3687
Kinase K D - K D - K - K - K - K - K - K - K
MBP tag
Substrate
