# Supplementary material for: M. tuberculosis Ser/Thr Protein Kinase D Phosphorylates an Anti-Anti–Sigma Factor Homolog
Source: PLoS Pathog. 2007 Apr 6;3(4):e49. doi: 10.1371/journal.ppat.0030049 (PMC1847690; doi:10.1371/journal.ppat.0030049)
Supplement: Supplementary file 4 [file ppat.0030049.sg004.pdf]

Figure S4
(A)

Protein	Residues	Sequence	Organism
**RsbV**	**51-63**	V S Y M D S T G L G V	*B. subtilis*
**BldG**	**52-64**	V D F L D S T G L G V	*S. coelicolor*
**Slr1859**	**52-64**	L E Y M S S A G L R I	*Synechocystis* PCC 6803
**Slr1856**	**49-61**	V P Y M S S A G L R L	*Synechocystis* PCC 6803
**SpoIIAA**	**53-65**	L S F M D S S G L G V	*B. subtilis*
**RsbR**	**200-212**	V P V V D T M V A H H	*B. subtilis*
**RsbS**	**54-66**	V D M I D S F I A K V	*B. subtilis*
**0516c**	**76-88**	L S H F S G A G I S L	*M. tuberculosis*

(B)

Family Member	Sequence
**Rv1904**	M G S C A Y A
**Rv2638**	M G C C A F A
**Rv1365c**	M G C C A V A
**Rv0941c**	M P V Y P T E
**Rv0516c**	F S G A G I S
**Rv3687c**	L G S V G L K
**SpoIIAA**	M D S S G L G
**RsbV**	M D S T G L G
