# Supplementary material for: M. tuberculosis Ser/Thr Protein Kinase D Phosphorylates an Anti-Anti–Sigma Factor Homolog
Source: PLoS Pathog. 2007 Apr 6;3(4):e49. doi: 10.1371/journal.ppat.0030049 (PMC1847690; doi:10.1371/journal.ppat.0030049)
Supplement: Supplementary file 5 [file ppat.0030049.st001.doc]

**SUPPORTING TABLE**

Table S1

|  | **wildtype kinase overexpression** | | | | | **inactive kinase overexpression** | | | | |
| --- | --- | --- | --- | --- | --- | --- | --- | --- | --- | --- |
| *hours post-induction* | *0* | *2* | *4* | *8* | *24* | *0* | *2* | *4* | *8* | *24* |
|  |  |  |  |  |  |  |  |  |  |  |
| *PE_PGRS7* | 1 | 1.16 | 1.22 | 1.31 | 1.33 | 1 | 1.11 | 1.82 | 1.25 | 0.62 |
| *mmpL3* | 1 | 1.19 | 1.65 | 1.16 | 1.02 | 1 | 1.2 | 1.74 | 1.68 | 0.98 |
| *Rv0580c* | 1 | 0.96 | 1.77 | 0.86 | 0.91 | 1 | 1.09 | 3.12 | n.d. | 0.72 |
| *PE_PGRS25* | 1 | 0.98 | 1.06 | 0.93 | 0.96 | 1 | 1.18 | 1.78 | 1.29 | 0.82 |
| *Rv0364* | 1 | n.d. | n.d. | 0.62 | 0.74 | 1 | 2.57 | 1.27 | 0.67 | 0.79 |
| *leuC* | 1 | 0.85 | n.d. | 0.9 | n.d. | 1 | 2 | 1.65 | 0.68 | 0.76 |
| *Rv3049c* | 1 | 0.95 | n.d. | 0.8 | 0.58 | 1 | 1.95 | 1.83 | n.d. | 0.82 |
| *Rv3412* | 1 | 0.8 | 0.64 | 0.76 | 0.65 | 1 | 1.8 | 1.55 | 0.84 | 0.78 |
| *Rv1682* | 1 | 0.86 | 0.7 | 0.95 | 1.09 | 1 | 1.13 | 1.91 | 1.06 | 1.08 |
| *Rv3364c* | 1 | 1.11 | 0.88 | 0.97 | 1.16 | 1 | 1.36 | 1.71 | 0.78 | 1.06 |
| *rplL* | 1 | 0.93 | 1.15 | 0.97 | 1.29 | 1 | 0.87 | 2.16 | 1.06 | 1.53 |
| *phoS1* | 1 | 0.93 | 0.91 | 0.95 | 1.25 | 1 | 0.85 | 1.55 | 1.16 | 1.78 |
| *atpB* | 1 | 0.77 | 0.78 | 1.06 | 1.42 | 1 | 0.8 | 1.06 | 0.93 | 1.87 |
| *atpF* | 1 | 0.73 | 0.89 | 0.85 | 1.49 | 1 | 0.85 | 1.18 | 1.13 | 2.41 |
| *atpA* | 1 | 0.78 | 0.87 | 0.97 | 1.43 | 1 | 0.91 | 1.1 | 1.18 | 2.01 |
| *Rv1792* | 1 | 0.9 | 1.16 | 0.89 | n.d. | 1 | 0.98 | 1.25 | 0.98 | 1.77 |
| *apt* | 1 | 0.91 | 1.45 | 0.91 | 1.05 | 1 | 1.06 | 1.26 | 1.03 | 1.78 |
| *PPE38* | 1 | 1.05 | 1.37 | 0.92 | 1.07 | 1 | 0.99 | 1.17 | 0.8 | 1.78 |
| *kasA* | 1 | 0.97 | 1.22 | 0.88 | 1.3 | 1 | 0.95 | 0.99 | 1.03 | 2.11 |
| *efpA* | 1 | 1.28 | 1.18 | 0.97 | 1.34 | 1 | 1.07 | 1.1 | 1.12 | 2.08 |
| *Rv1158c* | 1 | 0.86 | 0.85 | 0.58 | 1.04 | 1 | 0.74 | 0.75 | 0.74 | 1.75 |
| *Rv1566c* | 1 | 0.72 | 0.77 | 0.54 | n.d. | 1 | 0.77 | 0.9 | 0.94 | 2.28 |
| *Rv2147c* | 1 | 0.88 | n.d. | 0.6 | 0.69 | 1 | 0.55 | 0.97 | 0.94 | 1.54 |
| *Rv2908c* | 1 | 0.65 | 1.12 | 0.82 | 0.87 | 1 | 0.64 | 1.13 | n.d. | 1.72 |
| *cysG* | 1 | 0.77 | 0.74 | 1.08 | 1.04 | 1 | 1.12 | 0.81 | 1.99 | 1.24 |
| *gpsI* | 1 | 0.97 | 0.8 | 1.04 | 0.99 | 1 | 1.15 | 0.66 | 1.85 | 1.03 |
| *ahpE* | 1 | 0.93 | n.d. | 0.96 | 1.43 | 1 | 1.57 | 0.56 | n.d. | 1.37 |
| *rplP* | 1 | 0.91 | 0.73 | 1.03 | 0.91 | 1 | 1.14 | 1.25 | 1.92 | 1.13 |
| *rpmE* | 1 | 0.86 | 0.77 | 1.21 | 0.97 | 1 | 0.95 | 1.33 | 1.87 | 1.13 |
| *mmpS5* | 1 | 1.03 | 0.92 | 1.27 | n.d. | 1 | 1.04 | 1.47 | 1.93 | 1.15 |
| *Rv2161c* | 1 | 1.14 | 0.94 | 1.07 | 1.13 | 1 | 1.09 | 1.32 | 1.78 | 1.01 |
| *rpsS* | 1 | 0.99 | 0.74 | 0.94 | 1.13 | 1 | 0.68 | 1.13 | 1.8 | 1.51 |
| *moeY* | 1 | 0.94 | n.d. | 0.99 | n.d. | 1 | 0.85 | 1.22 | 1.89 | 1.62 |
| *rplW* | 1 | 0.86 | 0.73 | 0.95 | 1.05 | 1 | 0.71 | 0.97 | 2.01 | 1.26 |
| *rplQ* | 1 | 0.83 | 0.66 | 0.85 | 1.08 | 1 | 0.62 | 1.16 | 1.74 | 1.23 |
| *rpsG* | 1 | 0.93 | 0.86 | 0.88 | 1.17 | 1 | 1.04 | 0.96 | 1.72 | 1.48 |
| *Rv3747* | 1 | 0.93 | 0.6 | 0.69 | n.d. | 1 | 0.77 | 1.02 | n.d. | 2.36 |
| *aceE* | 1 | 1.01 | 0.68 | 0.87 | 1.34 | 1 | 1.09 | 1.16 | 1.73 | 1.29 |
| *rplD* | 1 | 0.82 | 0.84 | 1.17 | 1.05 | 1 | 1.61 | 1.13 | 2 | 0.97 |
| *Rv1615* | 1 | 0.98 | n.d. | 0.77 | 0.71 | 1 | 1.74 | 1.08 | 1.66 | 1.16 |
| *nadC* | 1 | 0.82 | 0.88 | 0.81 | 0.9 | 1 | 1.57 | 1.03 | 1.93 | 1.25 |
| *mrsA* | 1 | 1.02 | 0.74 | 0.75 | n.d. | 1 | 1.58 | n.d. | 2.64 | 1.27 |
| *rho* | 1 | 0.9 | 0.66 | 1.16 | 1.03 | 1 | 1.62 | 0.96 | 2.13 | 1.53 |
| *Rv1713* | 1 | 1.04 | 0.64 | 1.36 | 1.08 | 1 | 1.79 | 0.86 | 1.4 | 1.29 |
| *ruvC* | 1 | 0.81 | 0.72 | 0.88 | 1.19 | 1 | 1.77 | 1.13 | 1.28 | 1.21 |
| *PE_PGRS54* | 1 | 0.93 | 0.79 | 1.12 | 1.13 | 1 | 1.84 | 1.06 | 1.44 | 1.32 |
| *Rv0079* | 1 | 1.21 | 0.97 | 1.09 | 1.26 | 1 | 1.39 | 1.04 | 1.06 | 1.73 |
| *Rv0370c* | 1 | 1.11 | 0.93 | n.d. | 1.16 | 1 | 1.87 | 1.18 | 0.9 | n.d. |
| *Rv0801* | 1 | 1.25 | 0.85 | 1.09 | 1.27 | 1 | 1.72 | 0.91 | 1.34 | 1.49 |
| *folP2* | 1 | 1.2 | n.d. | 0.87 | 1.09 | 1 | 1.83 | 0.91 | 1.21 | 1.73 |
| *Rv1209* | 1 | 1.2 | 0.72 | 0.88 | 1.08 | 1 | 1.71 | 1.02 | 1.19 | 1.38 |
| *fdxC* | 1 | 1.21 | 0.82 | n.d. | 0.8 | 1 | 1.41 | 1.46 | 1.97 | 1.71 |
| *Rv1772* | 1 | 1.02 | 1.33 | 0.89 | 0.73 | 1 | 1.23 | 1.28 | 1.55 | 1.8 |
| *PPE10* | 1 | 0.9 | 1.08 | 0.83 | 1.02 | 1 | 1.73 | 1.29 | 1.26 | 1.77 |
| *Rv0460* | 1 | 0.93 | n.d. | 0.75 | 1.03 | 1 | 1.85 | 1.28 | 1.07 | 1.66 |
| *Rv3851* | 1 | 0.69 | n.d. | n.d. | 1.01 | 1 | 2.1 | 1.37 | 1.04 | 1.68 |
| *Rv2979c* | 1 | 1.07 | n.d. | 0.78 | 0.84 | 1 | 1.78 | 1.36 | 1.13 | n.d. |
| *lprK* | 1 | 1.03 | 0.78 | 0.93 | 1.04 | 1 | 1.71 | 1.26 | 1.38 | 1.89 |
| *aroP2* | 1 | 0.95 | 0.74 | 0.94 | 0.96 | 1 | 2.17 | 1.59 | 1.75 | 1.74 |
| *Rv2693c* | 1 | 0.99 | n.d. | 0.94 | 1.07 | 1 | 1.79 | 1.4 | 1.61 | 1.49 |
| *Rv1828* | 1 | 0.87 | 0.76 | 0.92 | 1.02 | 1 | 1.73 | 1.71 | 1.4 | 1.46 |
| *Rv3920c* | 1 | 0.97 | 0.7 | 1.1 | 1.04 | 1 | 1.69 | 1.45 | 2.14 | 1.93 |
| *Rv2260* | 1 | 0.66 | n.d. | 0.69 | 0.92 | 1 | 2.16 | 1.66 | 1.45 | n.d. |
| *Rv2792c* | 1 | 0.76 | 0.56 | 0.73 | 1.06 | 1 | 1.92 | 1.66 | 1.56 | 1.78 |
| *hupB* | 1 | 0.86 | 0.73 | 0.86 | 1.08 | 1 | 1.55 | 1.55 | 1.31 | 1.85 |
| *MT0450* | 1 | 0.82 | 0.66 | 0.69 | 0.94 | 1 | 1.92 | 1.82 | 1.09 | 1.53 |
| *PE_PGRS27* | 1 | 0.95 | n.d. | 0.9 | 0.98 | 1 | 2.04 | 2.2 | 1.05 | 1.6 |
| *Rv3582c* | 1 | 0.86 | 0.78 | 1.04 | 1.18 | 1 | 1.77 | 1.73 | n.d. | 1.4 |
| *atpE* | 1 | 0.76 | 0.75 | 1.06 | 1.39 | 1 | 1.22 | 1.07 | 1.39 | 2.77 |
| *Rv1303* | 1 | 0.84 | 0.7 | 1.06 | 1.12 | 1 | 1.38 | 0.86 | 1.56 | 2.5 |
| *atpH* | 1 | 0.73 | 0.68 | 0.96 | 1.2 | 1 | 1.49 | 1.04 | 1.56 | 2.5 |
| *fabD* | 1 | 0.93 | 0.69 | 0.71 | 1.35 | 1 | 1.33 | 1.21 | 1.79 | 2.66 |
| *Rv1884c* | 1 | 0.88 | 0.84 | 1.23 | 1.26 | 1 | 1.17 | 1.38 | 1.03 | 2.28 |
| *PE_PGRS57* | 1 | 0.89 | 0.97 | 1.15 | 1.29 | 1 | 1.42 | 1.27 | 1.21 | 1.84 |
| *acpM* | 1 | 0.7 | 0.65 | 1.04 | 1.79 | 1 | 1.35 | 1.56 | 1.64 | 2.85 |
| *Rv3482c* | 1 | 0.85 | 0.85 | 1.06 | 1.06 | 1 | 1.29 | 1.45 | 1.46 | 1.93 |
| *trpA* | 1 | 0.97 | 0.74 | 0.84 | 1.15 | 1 | 1.84 | 0.97 | 2.11 | 2.27 |
| *lipR* | 1 | 1.06 | 0.99 | 1.01 | 1.33 | 1 | 1.71 | 1.13 | 1.99 | 1.84 |
| *Rv1543* | 1 | 0.8 | 0.94 | 0.77 | 1.08 | 1 | 1.68 | 1.31 | 1.51 | 2.06 |
| *Rv2216* | 1 | 0.9 | n.d. | 0.84 | 1.39 | 1 | 1.54 | 1.28 | n.d. | 1.93 |
| *nuoJ* | 1 | 0.92 | 1.21 | 0.9 | 1.36 | 1 | 1.66 | 1.33 | 2.07 | 2.43 |
| *nuoL* | 1 | 0.88 | 1.03 | 0.94 | 1.19 | 1 | 1.6 | 1.12 | 1.4 | 1.95 |
| *Rv1883c* | 1 | 1.16 | 1.04 | 1.06 | 1.13 | 1 | 1.52 | 1.61 | 1.75 | 2.14 |
| *Rv3413c* | 1 | 1.08 | 0.99 | 0.92 | 1.17 | 1 | 1.36 | 1.43 | 1.99 | 2.5 |
| *Rv2625c* | 1 | 1.37 | 0.83 | 1.06 | 1.27 | 1 | 1.43 | 1.22 | 1.79 | 2.17 |
| *pks16* | 1 | 0.97 | 0.97 | 0.62 | 1.21 | 1 | 0.88 | 1.06 | 1.34 | 2.16 |
| *fbpC2* | 1 | 0.85 | 0.76 | 0.6 | 0.86 | 1 | 1.12 | 1.04 | 1.21 | 2.62 |
| *Rv1157c* | 1 | 0.96 | 0.87 | 0.64 | 1.06 | 1 | 1.22 | 1.06 | 1.06 | 2.3 |
| *Rv3005c* | 1 | 0.95 | 0.9 | 0.81 | 1.11 | 1 | 1.27 | 1.06 | 1.14 | 1.87 |
| *Rv1592c* | 1 | 0.67 | 1.01 | 0.59 | 1.21 | 1 | 1.27 | 1.1 | 1.66 | 3.05 |
| *tpx* | 1 | 0.65 | 1.18 | 0.79 | 0.94 | 1 | 1.21 | 0.99 | 1.65 | 2.14 |
| *ilvC* | 1 | 0.78 | 1.05 | 0.7 | 1.17 | 1 | 1.11 | 1.48 | 1.48 | 1.87 |
| *rpsF* | 1 | 0.88 | 0.67 | 0.75 | 0.86 | 1 | 1.26 | 0.81 | 1.96 | 1.95 |
| *Rv1201c* | 1 | n.d. | 0.73 | 0.75 | 0.95 | 1 | 1.29 | 0.86 | 1.88 | 1.69 |
| *trpC* | 1 | 0.93 | 0.62 | 0.77 | 0.95 | 1 | 1.39 | 0.75 | 2.16 | 1.72 |
| *bioB* | 1 | 0.87 | n.d. | 0.76 | 0.81 | 1 | 0.93 | 0.84 | 1.88 | 1.56 |
| *rpsR* | 1 | 0.93 | n.d. | 0.87 | 0.7 | 1 | 1.29 | 0.79 | n.d. | 1.83 |
| *Rv2721c* | 1 | 0.86 | 0.81 | 0.84 | 0.89 | 1 | 1.29 | 0.87 | 1.13 | 2.01 |
| *PE13* | 1 | 1.21 | 0.76 | 0.76 | 0.81 | 1 | 1.23 | 0.84 | 1.36 | 2.3 |
| *Rv2837c* | 1 | 0.99 | 0.92 | 0.87 | 0.86 | 1 | 1.32 | n.d. | 1.41 | 1.72 |
| *ppiA* | 1 | 0.9 | 0.62 | 0.81 | 0.96 | 1 | 1.29 | 1.04 | 1.66 | 2.19 |
| *rpsM* | 1 | 0.83 | 0.84 | 0.86 | 0.94 | 1 | 1.08 | 1.06 | 1.31 | 1.72 |
| *Rv3587c* | 1 | 0.79 | 0.59 | 0.62 | 0.81 | 1 | 1.21 | 0.85 | 1.57 | 1.88 |
| *MT2421* | 1 | 0.91 | 0.64 | 0.62 | 0.8 | 1 | 1.32 | 0.93 | 1.67 | 2.48 |
| *sodA* | 1 | 0.85 | 0.62 | 0.59 | 0.74 | 1 | 1.25 | 1.69 | 1.67 | 2.77 |
| *hns* | 1 | 0.96 | 0.82 | 0.8 | 0.83 | 1 | 1.26 | 1.27 | 1.57 | 1.83 |
| *Rv2450c* | 1 | 0.59 | 0.52 | 0.75 | 1.06 | 1 | 1.2 | 0.95 | 1.31 | 1.65 |
| *rpoA* | 1 | 0.61 | 0.61 | 0.8 | 0.84 | 1 | 1.27 | 1.13 | 1.72 | 1.73 |
| *Rv3583c* | 1 | 0.76 | 0.54 | 0.73 | 0.66 | 1 | 1.53 | 0.92 | 1.84 | 1.64 |
| *PPE68* | 1 | 0.5 | 0.76 | 0.7 | 0.74 | 1 | 1.59 | 1.04 | 1.31 | 1.43 |
| *gcvH* | 1 | 0.74 | 0.58 | 0.77 | 1.03 | 1 | 0.95 | 1.65 | 0.7 | 1.29 |
| *sahH* | 1 | 0.53 | 0.58 | 0.57 | 0.83 | 1 | 1.06 | 1.54 | 0.9 | 1.27 |
| *lpqE* | 1 | 0.57 | 0.49 | 0.54 | 0.74 | 1 | 1.15 | 1.79 | 0.94 | 1.56 |
| *Rv1518* | 1 | 0.62 | n.d. | 0.48 | 1.04 | 1 | 1.52 | 1.19 | 0.92 | 2.04 |
| *Rv3224* | 1 | 0.76 | 0.53 | 0.72 | 1.12 | 1 | 1.14 | 1.25 | 1.19 | 1.71 |
| *mtrA* | 1 | 0.68 | 0.52 | 0.71 | 1.08 | 1 | 1.27 | 1.54 | 0.97 | 1.93 |
| *MT2420* | 1 | 0.71 | 0.5 | 0.63 | 0.98 | 1 | 1.31 | 1.82 | 1.72 | 1.97 |
| *fadE8* | 1 | 1.14 | 2.11 | 0.82 | 0.82 | 1 | 1.13 | 0.78 | 0.93 | 0.79 |
| *pyrB* | 1 | n.d. | 1.74 | 0.78 | 1.09 | 1 | 1.36 | 0.94 | 1.16 | n.d. |
| *metB* | 1 | 0.85 | 1.72 | 0.69 | 1.33 | 1 | 0.78 | 0.78 | n.d. | 1.23 |
| *ctaD* | 1 | 0.96 | 1.73 | 0.81 | 1.39 | 1 | 0.66 | 0.89 | n.d. | 1.13 |
| *rplX* | 1 | 0.9 | 3.61 | 1.01 | 1.09 | 1 | 0.82 | 0.72 | n.d. | 0.84 |
| *Rv2554c* | 1 | 1.08 | 2.16 | 1.31 | n.d. | 1 | 0.68 | 0.86 | 1.3 | n.d. |
| *Rv2188c* | 1 | 1.09 | 1.78 | 0.85 | 1.3 | 1 | 0.93 | 0.85 | 0.86 | 0.71 |
| *MT1356* | 1 | 1.13 | 2.66 | 1.09 | 2.55 | 1 | 0.83 | 0.73 | 0.76 | 0.99 |
| *Rv0666* | 1 | 1.01 | 1.84 | 1.07 | 0.99 | 1 | 1.27 | 1.37 | 0.88 | 1.27 |
| *Rv1000* | 1 | 1.14 | 2.53 | 1.13 | n.d. | 1 | 1.51 | 2.03 | n.d. | 1.65 |
| *Rv1006* | 1 | 1.27 | 1.89 | 1.36 | 1.37 | 1 | 1.06 | 1.28 | 0.94 | 1.33 |
| *rnhB* | 1 | 1.25 | 1.83 | 1.45 | 1.43 | 1 | 1.16 | 1.33 | 0.82 | 1.57 |
| *PE_PGRS31* | 1 | 1.27 | 1.8 | 1.09 | n.d. | 1 | 1.09 | 1.23 | n.d. | 1.42 |
| *fdhF* | 1 | 1.19 | 2.2 | 1.32 | 1.21 | 1 | 1.03 | 1.27 | 0.85 | 1.53 |
| *cysH* | 1 | 1.07 | 2 | 1.04 | 1.25 | 1 | 1.12 | 1.09 | 0.96 | 1.39 |
| *murX* | 1 | 1.11 | 1.75 | 1.1 | 1.37 | 1 | 0.93 | 1.21 | 1.02 | 1.07 |
| *secF* | 1 | 1.31 | 1.85 | 1.16 | n.d. | 1 | 1.08 | 1.15 | n.d. | 1.06 |
| *Rv3472* | 1 | 1.3 | 1.93 | 1.21 | 1.25 | 1 | 0.93 | 1.16 | 0.91 | 0.86 |
| *PPE60* | 1 | 1.13 | 1.99 | 1.15 | 1.17 | 1 | 1.17 | 0.95 | 1.31 | 1.31 |
| *Rv2164c* | 1 | 0.88 | 1.84 | 0.93 | 1.25 | 1 | 0.89 | 1.11 | 1.08 | 1.36 |
| *Rv3734c* | 1 | 1.06 | 1.77 | 0.8 | 1.17 | 1 | 1.12 | 1.27 | 1.05 | 1.4 |
| *Rv2326c* | 1 | 1.4 | 2.08 | 1.96 | n.d. | 1 | 1.38 | 1.05 | 1.23 | n.d. |
| *Rv2081c* | 1 | 1.47 | 1.72 | 1.65 | 1.57 | 1 | 0.93 | 1.3 | 1.22 | 0.97 |
| *Rv3879c* | 1 | 1.24 | 1.53 | 1.97 | 1.22 | 1 | 1.05 | 1.27 | 1.54 | 0.9 |
| *Rv3085* | 1 | 1.59 | 1.13 | 1.27 | 1.57 | 1 | 1.21 | 1.14 | 1.79 | 1.2 |
| *Rv2010* | 1 | 1.41 | n.d. | 1.03 | 0.58 | 1 | 0.99 | 0.75 | 0.8 | n.d. |
| *Rv1671* | 1 | 1.08 | 0.74 | 1.04 | 0.97 | 1 | 0.95 | 0.76 | 0.96 | 0.55 |
| *Rv1870c* | 1 | 0.99 | 0.77 | 1.17 | 0.76 | 1 | 0.76 | n.d. | 0.95 | 0.53 |
| *Rv0748* | 1 | 0.83 | 0.77 | 0.84 | 0.73 | 1 | 1.16 | 1.39 | 0.7 | 0.37 |
| *Rv3613c* | 1 | 0.94 | 0.85 | 0.88 | 0.93 | 1 | 0.86 | 1.04 | 0.92 | 0.47 |
| *Rv0282* | 1 | 0.97 | 1.08 | 0.83 | 0.58 | 1 | 0.86 | 0.77 | 1.23 | 0.73 |
| *ideR* | 1 | 0.96 | n.d. | 0.78 | 0.52 | 1 | 1.09 | 0.93 | 1.24 | 0.59 |
| *35kd_ag* | 1 | 0.72 | 0.92 | 0.59 | 0.38 | 1 | 1.34 | 0.82 | 1.32 | 0.62 |
| *Rv0313* | 1 | 1.03 | n.d. | 0.82 | 0.67 | 1 | 1.2 | n.d. | n.d. | 0.52 |
| *Rv1462* | 1 | 1.01 | 1.4 | 0.74 | 0.66 | 1 | 1.13 | 1.01 | n.d. | 0.43 |
| *Rv1464* | 1 | 1.06 | n.d. | 0.86 | 0.67 | 1 | 1.03 | 1.04 | 1.13 | 0.5 |
| *Rv1465* | 1 | 1.01 | n.d. | 0.75 | 0.59 | 1 | 1.17 | 0.73 | n.d. | 0.36 |
| *Rv1783* | 1 | 0.86 | n.d. | 0.81 | 0.76 | 1 | 1.06 | n.d. | 1.03 | 0.46 |
| *Rv0049* | 1 | 1.09 | n.d. | 0.79 | 0.6 | 1 | 1.13 | 0.96 | 2.1 | n.d. |
| *Rv3837c* | 1 | 1.04 | n.d. | 0.71 | 0.52 | 1 | 1.08 | 1.08 | n.d. | 0.5 |
| *desA3* | 1 | 1.22 | 0.68 | 0.87 | 0.75 | 1 | 0.99 | 0.81 | 1.47 | 0.48 |
| *Rv3678c* | 1 | 0.97 | 0.97 | 0.95 | 0.86 | 1 | 0.91 | 0.81 | 1.13 | 0.58 |
| *MT1364* | 1 | 0.93 | 0.84 | 0.86 | 0.66 | 1 | 0.91 | 0.77 | 1.33 | 0.49 |
| *Rv0516c* | 1 | 1.01 | 0.82 | 0.41 | 0.18 | 1 | 1.32 | 1.06 | 0.52 | 0.42 |
| *Rv1461* | 1 | 0.93 | 0.87 | 0.62 | 0.53 | 1 | 1.24 | 1.19 | 0.77 | 0.72 |
| *Rv0298* | 1 | 0.8 | 0.59 | 0.69 | 0.55 | 1 | 1.24 | 1.35 | 0.73 | 0.56 |
| *clpP2* | 1 | 0.7 | 0.65 | 0.59 | 0.55 | 1 | 0.93 | 1.2 | 0.55 | 0.57 |
| *groEL2* | 1 | 0.49 | 0.91 | 0.43 | 0.46 | 1 | 0.94 | 1.14 | 0.81 | 0.47 |
| *groEL1* | 1 | 0.73 | 1.16 | 0.6 | 0.55 | 1 | 1.16 | 1.1 | 0.71 | 0.49 |
| *Rv1174c* | 1 | 0.81 | 0.98 | 0.73 | 0.62 | 1 | 0.91 | 1.13 | 0.9 | 0.51 |
| *groES* | 1 | 0.64 | 0.67 | 0.57 | 0.53 | 1 | 0.88 | 1.28 | 0.67 | 0.33 |
| *argJ* | 1 | 0.93 | 0.91 | 0.78 | 0.74 | 1 | 0.76 | 0.86 | 0.97 | 0.59 |
| *Rv0250c* | 1 | 0.81 | 0.8 | 0.61 | 0.26 | 1 | 1.05 | 0.91 | 0.5 | 0.3 |
| *sigB* | 1 | 0.8 | 0.89 | 0.7 | 0.32 | 1 | 0.74 | 0.81 | 0.59 | 0.24 |
| *hsp* | 1 | 0.66 | 0.58 | 0.24 | 0.11 | 1 | 0.63 | 0.76 | 0.55 | 0.11 |
| *Rv2111c* | 1 | 0.85 | n.d. | 0.72 | 0.43 | 1 | 0.72 | 0.94 | 0.94 | 0.51 |
| *clpP* | 1 | 0.76 | 0.56 | 0.49 | 0.41 | 1 | 0.72 | 0.83 | 0.92 | 0.38 |
| *MT1178* | 1 | 0.84 | 0.52 | 0.6 | 0.23 | 1 | 0.46 | 0.76 | 0.54 | 0.35 |
| *Rv2745c* | 1 | 0.77 | 0.51 | 0.51 | 0.33 | 1 | 1.09 | 0.57 | 0.85 | 0.37 |
| *Rv2302* | 1 | 1.04 | 0.48 | 0.87 | 0.66 | 1 | 0.84 | 0.94 | n.d. | 0.56 |
| *Rv2493* | 1 | 0.97 | n.d. | 0.76 | 0.57 | 1 | 0.82 | 0.87 | 0.84 | n.d. |
| *PE19* | 1 | 0.66 | 0.73 | 0.52 | 0.66 | 1 | 0.77 | 0.9 | 0.73 | 0.7 |
| *MT2558* | 1 | 0.68 | 0.62 | 0.58 | 0.49 | 1 | 0.73 | 0.75 | 0.69 | 0.66 |
| *MT2467* | 1 | 0.8 | 0.7 | 0.58 | 0.68 | 1 | 0.72 | 0.87 | 0.92 | 0.62 |
| *dnaK* | 1 | 0.67 | 0.69 | 0.48 | 0.47 | 1 | 0.63 | 0.65 | 0.32 | 0.24 |
| *dnaJ* | 1 | 0.75 | 0.62 | 0.62 | 0.59 | 1 | 0.84 | 0.72 | 0.4 | 0.28 |
| *grpE* | 1 | 0.77 | 1.16 | 0.55 | 0.52 | 1 | 0.81 | 0.58 | 0.35 | 0.35 |
| *Rv2053c* | 1 | 1.05 | 0.93 | 0.85 | 0.57 | 1 | 0.89 | 0.71 | 0.54 | 0.42 |
| *sec* | 1 | 0.21 | 0.23 | 0.15 | 0.08 | 1 | 0.14 | 0.09 | 0.06 | 0.06 |
| *pknD* | 1 | 0.2 | 0.19 | 0.18 | 0.1 | 1 | 0.13 | 0.08 | 0.04 | 0.04 |
| *Rv3848* | 1 | 0.33 | 0.19 | 0.14 | 0.07 | 1 | 0.29 | 0.13 | 0.08 | 0.13 |
| *MT2466* | 1 | 0.85 | 0.75 | 0.63 | 0.53 | 1 | 0.68 | 0.71 | 0.51 | 0.59 |
| *Rv0571c* | 1 | 1.12 | 1.02 | 0.97 | 0.78 | 1 | 0.76 | 0.54 | 0.39 | 0.37 |
| *Rv0144* | 1 | 1.06 | 0.97 | 0.92 | 0.9 | 1 | 0.64 | 0.63 | 0.57 | 0.55 |
| *Rv2082* | 1 | 0.97 | n.d. | 0.81 | n.d. | 1 | 0.5 | 0.54 | 0.27 | 0.37 |
| *Rv1405c* | 1 | 0.57 | 0.47 | 0.41 | n.d. | 1 | 0.29 | 0.16 | 0.12 | 0.14 |
| *Rv2778c* | 1 | 0.67 | 0.57 | 0.62 | 0.31 | 1 | 0.34 | 0.21 | 0.13 | 0.18 |
| *Rv0431* | 1 | 1.07 | n.d. | 0.66 | 0.74 | 1 | 0.55 | 0.44 | 0.29 | 0.31 |
| *Rv2082* | 1 | 0.89 | n.d. | 0.61 | n.d. | 1 | 0.62 | 0.42 | 0.31 | 0.31 |
| *hspR* | 1 | 0.9 | 1.55 | 0.84 | 0.76 | 1 | 0.79 | 0.53 | n.d. | 0.38 |
| *Rv3880c* | 1 | 1.01 | 1.16 | 1.06 | 0.84 | 1 | 0.69 | 0.7 | 0.81 | 0.58 |
| *Rv0493c* | 1 | 1.03 | 0.88 | 0.93 | 1.01 | 1 | 0.72 | 0.82 | 0.83 | 0.5 |
| *argD* | 1 | 0.91 | 0.93 | 0.85 | 0.97 | 1 | 0.77 | 0.75 | 0.84 | 0.55 |
| *MT1585.1* | 1 | 0.97 | 0.98 | 0.75 | 0.8 | 1 | 0.59 | 0.66 | 0.65 | 0.36 |
| *Rv2557* | 1 | 0.91 | 0.99 | 0.75 | 1.14 | 1 | 0.82 | 0.69 | 0.78 | 0.54 |
| *Rv3289c* | 1 | 1.03 | 0.63 | 1.01 | 1.04 | 1 | 0.91 | 0.79 | 0.56 | 0.6 |
| *MT3282* | 1 | 0.97 | 0.93 | 1.44 | 1.1 | 1 | 0.76 | 0.69 | 0.69 | 0.57 |
| *galE2* | 1 | 1.39 | 0.93 | 0.94 | 1.16 | 1 | 0.53 | 0.62 | 0.63 | n.d. |
| *bcp* | 1 | 1.1 | 0.89 | 0.98 | 1.1 | 1 | 0.6 | 0.72 | 0.53 | 0.73 |
| *Rv3213c* | 1 | 0.97 | 0.98 | 0.75 | 1.16 | 1 | 0.66 | 0.58 | n.d. | 0.77 |
| *Rv3805c* | 1 | 0.87 | 1.46 | 0.81 | 1.1 | 1 | 0.54 | 0.62 | 0.47 | 0.71 |
| *PPE51* | 1 | 0.96 | 1.49 | 0.97 | 1.05 | 1 | 0.7 | 0.97 | 0.7 | 0.5 |
| *argB* | 1 | 0.93 | 0.97 | 0.95 | 0.96 | 1 | 0.57 | 1.07 | 0.93 | 0.72 |
| *Rv2185c* | 1 | 0.76 | n.d. | 0.69 | n.d. | 1 | 0.54 | 0.79 | 0.77 | 0.75 |
| *Rv2573* | 1 | 0.93 | 0.81 | 0.81 | 1.19 | 1 | 0.55 | 0.84 | 0.8 | 0.85 |
| *accA3* | 1 | 0.77 | 0.76 | 0.58 | 1.16 | 1 | 0.63 | 0.74 | 0.76 | 0.99 |
| *Rv2376c* | 1 | 0.76 | 1.07 | 0.67 | 0.94 | 1 | 0.58 | 0.97 | 0.96 | 0.95 |
| *rpmG-1* | 1 | 0.71 | 0.88 | 0.68 | 0.88 | 1 | 0.57 | 0.86 | n.d. | 1.09 |
| *katG* | 1 | 0.7 | 0.99 | 1.04 | 1.39 | 1 | 1.2 | 1.01 | 0.49 | 1.43 |
| *Rv2261c* | 1 | 1.1 | 1 | 0.66 | 1.49 | 1 | 1.19 | 1.13 | 0.54 | n.d. |
| *Rv1072* | 1 | n.d. | 1.04 | 0.78 | 0.84 | 1 | 1.31 | 0.98 | 0.54 | 0.64 |
| *nrdI* | 1 | 1.04 | 0.89 | 0.81 | 0.98 | 1 | 1.25 | 0.81 | 0.53 | 0.77 |
| *lprB* | 1 | 0.81 | n.d. | 0.8 | 0.9 | 1 | 1.04 | 0.97 | 0.58 | 0.81 |
| *Rv3828c* | 1 | 0.81 | 0.58 | 0.69 | 0.78 | 1 | 1.06 | 1.03 | 0.56 | 0.74 |
| *Rv1830* | 1 | 1 | 0.75 | n.d. | 1.01 | 1 | 1.06 | 0.88 | 0.51 | 0.97 |
| *recA* | 1 | 0.95 | 0.86 | 0.8 | 0.97 | 1 | 0.91 | 0.8 | 0.5 | 1.33 |
| *Rv2449c* | 1 | 0.99 | 0.76 | 1.25 | 1.12 | 1 | 1.16 | 0.58 | 0.79 | 0.97 |
| *Rv1435c* | 1 | 0.9 | 0.76 | 0.62 | 0.87 | 1 | 0.99 | 0.55 | n.d. | 0.91 |
| *rplO* | 1 | 0.81 | 0.68 | 0.87 | 0.97 | 1 | 0.58 | 0.82 | 1.12 | 0.78 |
| *rplU* | 1 | 1.01 | 0.59 | 1.04 | 0.76 | 1 | 0.66 | n.d. | 1.24 | 0.89 |
| *sigA* | 1 | 0.77 | 0.58 | 0.75 | 0.74 | 1 | 0.62 | 0.75 | 1.31 | 0.82 |
| *rpsO* | 1 | 0.67 | 0.5 | n.d. | 0.54 | 1 | 0.63 | 0.62 | n.d. | 0.9 |
| *Rv0635* | 1 | 0.78 | 0.55 | 0.76 | 0.97 | 1 | 0.88 | 0.93 | 1.21 | 1.07 |
| *rplJ* | 1 | 0.74 | 0.54 | 0.66 | n.d. | 1 | 0.75 | 0.87 | n.d. | 1.18 |
| *rplA* | 1 | 0.74 | 0.57 | 0.85 | 0.9 | 1 | 0.81 | 0.76 | 1.65 | 1.13 |
| *PE5* | 1 | 0.9 | 0.8 | 0.57 | 0.68 | 1 | 1.09 | 1.01 | 1.03 | 1.04 |
| *Rv0287* | 1 | 0.83 | 0.8 | 0.58 | 0.68 | 1 | 0.94 | 0.99 | 0.91 | 0.95 |
| *esxS* | 1 | 0.78 | 0.6 | 0.55 | 0.51 | 1 | 0.87 | 0.96 | 0.97 | 0.97 |
| *Rv2050* | 1 | 0.57 | 0.55 | 0.52 | 0.46 | 1 | 0.62 | n.d. | n.d. | 0.68 |
| *Rv3891c* | 1 | 0.69 | 0.64 | 0.72 | 0.52 | 1 | 0.67 | 0.97 | 1.34 | 0.93 |
| *nrdF* | 1 | 0.77 | 0.56 | 0.76 | 0.7 | 1 | 0.94 | 1.09 | 1.2 | 0.91 |
| *fixA* | 1 | 0.74 | 0.59 | 0.62 | 0.76 | 1 | 0.78 | 1.15 | 1.3 | 1.06 |
| *PPE50* | 1 | 0.48 | 0.35 | 0.4 | 0.46 | 1 | 0.73 | 0.95 | 1.35 | 1.14 |
| *Rv1987* | 1 | 0.76 | 0.54 | 0.69 | 0.61 | 1 | 0.92 | 0.93 | 1.31 | 1.28 |
| *trxC* | 1 | 0.74 | 0.61 | 0.65 | 0.55 | 1 | 1.12 | 0.94 | 1.16 | 1.11 |
| *rpmJ* | 1 | 0.54 | 0.55 | 0.57 | 0.7 | 1 | 1.24 | 0.97 | 1.34 | 1.14 |
| *infA* | 1 | 0.69 | 0.58 | 0.66 | 0.63 | 1 | 1.17 | 1.08 | 1.47 | 1.26 |
| *Rv2791c* | 1 | 0.73 | 0.5 | 0.87 | 0.7 | 1 | 1.3 | 0.71 | 1.16 | 0.91 |
| *whiB1* | 1 | 0.62 | 0.47 | 0.95 | 0.67 | 1 | 1.2 | 0.56 | 1.3 | 0.8 |
| *Rv2840c* | 1 | 0.59 | 0.52 | 0.75 | 0.74 | 1 | 0.99 | 0.86 | 1.27 | 0.79 |
| *bfrB* | 1 | 0.52 | 0.44 | 0.7 | 0.45 | 1 | 1.13 | 0.75 | 1.49 | 0.76 |
| *Rv2641* | 1 | 0.77 | 0.74 | 0.73 | 0.59 | 1 | 0.97 | 1.4 | 1.13 | 1.23 |
| *nrdE* | 1 | 0.77 | 0.61 | 0.7 | 0.65 | 1 | 1.23 | 1.77 | 1.58 | 1.27 |
| *nrdH* | 1 | 0.82 | 0.54 | 0.61 | 0.69 | 1 | 1.26 | 1.42 | 1.31 | 1.12 |
| *rplM* | 1 | 0.77 | 0.62 | 0.65 | 0.84 | 1 | 1.01 | 1.2 | 1.74 | 0.99 |
| *cspA* | 1 | 0.84 | 0.49 | 0.97 | 0.82 | 1 | 1.15 | 1.2 | 1.68 | 1.16 |
| *Rv2256c* | 1 | 0.97 | 0.49 | 0.72 | 0.76 | 1 | 1.37 | 1.01 | 0.9 | 0.71 |
| *Rv3220c* | 1 | 0.92 | 0.57 | 0.66 | 0.98 | 1 | 1.39 | 0.88 | n.d. | 1.09 |
| *Rv3221c* | 1 | 0.89 | 0.54 | 1.08 | 1.04 | 1 | 1.26 | 0.73 | 0.91 | 1.15 |
